# Planarians as a Model to Assess *In Vivo* the Role of Matrix Metalloproteinase Genes during Homeostasis and Regeneration

**DOI:** 10.1371/journal.pone.0055649

**Published:** 2013-02-06

**Authors:** Maria Emilia Isolani, Josep F. Abril, Emili Saló, Paolo Deri, Anna Maria Bianucci, Renata Batistoni

**Affiliations:** 1 Dipartimento di Biologia, Università di Pisa, Pisa, Italy; 2 Dipartimento di Scienze Farmaceutiche, Università di Pisa, Pisa, Italy; 3 Departament de Genètica, Universitat de Barcelona and Institut de Biomedicina de la Universitat de Barcelona, Barcelona, Catalonia, Spain; National Cancer Institute, United States of America

## Abstract

Matrix metalloproteinases (MMPs) are major executors of extracellular matrix remodeling and, consequently, play key roles in the response of cells to their microenvironment. The experimentally accessible stem cell population and the robust regenerative capabilities of planarians offer an ideal model to study how modulation of the proteolytic system in the extracellular environment affects cell behavior *in vivo*. Genome-wide identification of *Schmidtea mediterranea* MMPs reveals that planarians possess four *mmp*-like genes. Two of them (*mmp1* and *mmp2*) are strongly expressed in a subset of secretory cells and encode putative matrilysins. The other genes (*mt-mmpA* and *mt-mmpB*) are widely expressed in postmitotic cells and appear structurally related to membrane-type MMPs. These genes are conserved in the planarian *Dugesia japonica*. Here we explore the role of the planarian *mmp* genes by RNA interference (RNAi) during tissue homeostasis and regeneration. Our analyses identify essential functions for two of them. Following inhibition of *mmp1* planarians display dramatic disruption of tissues architecture and significant decrease in cell death. These results suggest that *mmp1* controls tissue turnover, modulating survival of postmitotic cells. Unexpectedly, the ability to regenerate is unaffected by *mmp1(RNAi).* Silencing of *mt-mmpA* alters tissue integrity and delays blastema growth, without affecting proliferation of stem cells. Our data support the possibility that the activity of this protease modulates cell migration and regulates anoikis, with a consequent pivotal role in tissue homeostasis and regeneration. Our data provide evidence of the involvement of specific MMPs in tissue homeostasis and regeneration and demonstrate that the behavior of planarian stem cells is critically dependent on the microenvironment surrounding these cells. Studying MMPs function in the planarian model provides evidence on how individual proteases work *in vivo* in adult tissues. These results have high potential to generate significant information for development of regenerative and anti cancer therapies.

## Introduction

The extracellular matrix (ECM) provides structural support for tissue organization and also relays environmental signals to cells, influencing their behavior [1], [2]. Tightly regulated remodeling of this structure occurs in a wide range of physiological processes, including tissue homeostasis and regeneration, and dysregulation can result in a wide range of pathological conditions [3], [4]. Understanding the interactions between cells and ECM is crucial to realize new therapies, as well as to improve scaffold design for regenerative medicine applications [5], [6].

Planarians (Platyhelminthes, Lophotrocozoa) are an invertebrate model that has great potential for elucidating how the dynamics of ECM remodeling influence the behavior of cells during homeostasis and regeneration. These worms are a particularly attractive system because exhibit tissue complexity and ECM characteristics that are considered ancestral in many respects [7], [8], [9]. In addition, planarians constantly turnover all tissues and can activate amazing regeneration capabilities [10], [11], [12]. Such remarkable developmental plasticity depends on a large population of adult stem cells, named neoblasts, distributed throughout the mesenchymal tissue (parenchyma) of the animal, except the region anterior to the photoreceptors and the pharynx [13]. Recent studies provide evidence that the planarian stem cell system is complex and hierarchically ordered, and includes pluripotent stem cells, progenitors and lineage-restricted stem cells that are characterized by specific transcriptional profiles [14], [15], [16]. Although stationary in intact animals, neoblasts become quickly mobilized following an injury and initiate an intense proliferation program, mediated by activation of a large set of wound-induced genes [17], [18]. Two distinct phases of mitotic responses occur during regeneration: an initial body-wide mitotic response to injury and a second phase of intense proliferation at the wound site, depending by tissue absence [19]. Local mitotic response of neoblasts gives rise to progenitors that migrate and differentiate into appropriate cell types, resulting in the formation of the regeneration blastema [15], [20]. This process, tightly coordinated with apoptosis-mediated cell death in the stump, recreates exactly the missing body parts, ensuring the correct proportions in the new worm [21], [22].

Ultrastructural studies show that planarian stem cells are surrounded by rich ECM and migrate through this structure [23], [24]. These observations suggest that the extracellular environment is critical to create the dynamic cues that guarantee growth, survival, differentiation and mobilization of stem cells. How dramatic remodeling of the ECM during regeneration and continuous homeostatic replacement may affect cell behavior is currently unknown. It has been demonstrated that when planarians receive damage-inducing treatments release collagen-degrading metalloproteinases (MMPs) [25]. This finding supports the possibility that cell-ECM interactions during regeneration of the planarian body involve MMPs.

In the current study, we report the first characterization of genes encoding candidate MMP proteins in two related planarian species that have become models of choice for the study of the molecular mechanisms that control regeneration: *Schmidtea mediterranea* and *Dugesia japonica* [26]. MMPs constitute a multi-gene family of structurally related zinc-dependent secreted or membrane-anchored proteinases, with a highly conserved modular structure [27], [28], [29]. The minimal MMP forms consist only of the pro-peptide and the catalytic domain. However, most of these enzymes are characterized by additional hemopexin-like repeats that are implicated in substrate specificity. Membrane-anchored MMPs (MT-MMPs) possess an additional transmembrane domain and also contain a furin-like cleavage site, which is important during enzyme activation [30], [31]. MMPs are synthesized as pro-enzymes and their activity may be regulated at different levels. A variety of regulatory mechanisms have been proposed based on *in vitro* studies [32], [33]. Tightly regulated transcription of *mmp* genes primarily contributes to modulate MMP function in different tissues [34], [35]. In general, expression of most MMPs tends to be quite low in normal tissues, but significant activation is detected in rapidly remodeling tissues or is associated with invasiveness of cancer cells [36], [37]. Known for the ability to catalyze the hydrolysis of most components of the ECM, MMPs also play an intriguing role on cell fate and behavior by influencing the remodeling of the cellular microenvironment and/or by altering intercellular signaling [38], [39]. It is becoming increasingly clear that elucidation of the basic mechanisms of MMPs action, especially *in vivo*, is essential for a coherent understanding of the dynamics of ECM remodeling [40], [41].

The number of MMPs varies considerably in diverse organisms. To date, more than 20 MMPs have been isolated in mammals [41]. Because of the high level of redundancy, mechanistic understanding of their functions *in vivo* has often been elusive in mammalian models [42], [43]. MMP-like enzymes have been identified in diverse invertebrate groups, including echinoderms, crustaceans, nematodes, and also in organisms as simple as *Hydra* [44], [45], [46], [47]. Among invertebrates most information comes from *Drosophila,* whose genome encodes only two MMPs - one secreted type and one membrane-anchored type [48], [49]. The limited number of *Drosophila* MMPs allowed precise dissection of the mechanisms of action and regulation of these enzymes *in vivo*, as well as identification of tissue sources [49].

Through a bioinformatic approach, we have identified four MMP-related genes in the planarian *S. mediterranea.* We demonstrate that these genes (*Smed-mmp1, Smed-mmp2*, *Smed-mt-mmpA* and *Smed-mt-mmpB*) have conserved counterparts in the related species *D. japonica* (*Dj-mmp1, Dj-mmp2*, *Dj-mt-mmpA* and *Dj-mt-mmpB*), with similar expression patterns and functions in the two species. This conservation probably reflects similar selective pressures that these planarians faced during evolution from a common platyhelminth ancestor. This conservation is in good agreement with the essential roles of these genes in intact (uninjured) worms and during regeneration, as demonstrated by our RNAi studies. In line with that reported for other organisms [28] our functional data point to a critical role for these MMPs in a variety of cellular activities, including proliferation, apoptosis and cell migration.

## Materials and Methods

### Animals

A clonal strain (BCN10) [50] of the asexual biotype of *S. mediterranea* and an asexual strain (GI) of the planarian *D. japonica* were used in this study. The animals were reared at 18°C in planarian artificial medium (*S. mediterranea*) or autoclaved stream water (*D. japonica*), and starved for at least a week before the experiments. For regeneration studies, animals were cut at a portion posterior to the auricles and/or between pharynx and tail. Lateral regeneration was obtained by sagittal sectioning of the animals. For heat shock treatment, intact planarians were maintained at 28°C for 20 hours before being harvested for RNA extraction. Thirty days-starved planarians were used for starvation analysis.

### Gene Cloning and Sequence Analysis

Genes encoding MMPs in other organisms were used to query the *S. mediterranea* genome [51] and four published transcript databases [52], [53], [54], [55], using NCBI-BLASTN and TBLASTX [56]. Four different *mmp* genes were identified and specific primers were used to amplify those sequences from cDNA. *S. mediterranea* primers were also used to amplify the counterparts in *D. japonica*. MMP domains were validated on the BLAST hits by searching on the PFAM domains database [57]. Structure prediction was assessed by SMART ([58]; http://smartembl-heidelbergde/). The pro-peptide cleavage site was predicted using SignalP 3.0 (http://www.cbs.dtu.dk/services/SignalP/). The presence of a membrane anchor domain and a furin cleavage site in SMED-MT-MMPA and SMED-MT-MMPB has been identified by http://www.cbs.dtu.dk/services/TMHMM/ [59] and http://www.cbs.dtu.dk/services/SignalP/, respectively. Translated amino acid sequences of planarian MMP candidate sequences were aligned against a set of 267 sequences with candidates across different taxonomic groups (ranging from 4 to 27 species, for MMP26 and MMP19 sub-families respectively), and different MMP protein sub-families (23 distinct annotated sets). Sequences for the sets of MMP protein sub-families were derived from the already available MMP clusters from NCBI-HomoloGene database [http://www.ncbi.nlm.nih.gov/homologene/]. Those clusters mainly contained vertebrate protein sequences, so that, in order to broaden the phylogenetic analysis with further species, homolog sequences for each subfamily were retrieved from NCBI-UniGene, RefSeq and UniProt databases [60], [61], [62]. The alignment of all MMP protein sequences was built using muscle [63]; alignment format conversions were performed when required by scripts using the module Bio::AlignIO from BioPerl [64]. Only 706 conserved positions, about 31% of the total length in 5 selected blocks, were considered for the phylogenetic analysis. Those positions were selected by Gblocks [65], where all gap positions were allowed, a minimum length of 10 aa for a block, a minimum of 136 sequences for a flanking and conserved positions, and a maximum of 50 contiguous non-conserved positions. After this filtering step, 14 sequences were identical; the duplicates were removed and this was annotated by merging the sequence identifiers and 259 sequences were left on the alignments for posterior analyses. Maximum-likelihood phylogenetic analysis was conducted with RAxML [66], under the PROTCATBLOSUM62 model; a bootstrap value of 500 was set. The corresponding tree was drawn using iTOL web server [67]. Hidden Markov models (HMMs) for five PFAM domains (Matrixin/PF00413, Hemopexin/PF00045, a putative Peptido-Glycan Binding domain PGB1/PF01471, Fibronectin type II/PF00040, and a Domain of Unknown Function DUF3377/PF11857), were mapped against all the protein MMP sequences with Hmmer [68]; the coordinates of the hits having an E-value greater than 10 e^−5^ were then processed to draw the domain structures around the leaves of the tree, also using iTOL. Finally, all identified genes and predicted proteins were named based on their structural motifs with the nomenclature proposed by [69]. *S. mediterranea* sequences were submitted to GenBank: *Smed-mmp1* (HE577118.1), *Smed-mmp2* (HE577119.1), *Smed-mt-mmpA* (HE577120.1), and *Smed-mt-mmpB* (HE998773.1). *Dj-mmp1* and *Dj-mt-mmpA* homologs were retrieved from the *D. japonica* EST sequences dataset published on [70].

### Irradiation

As neoblasts are the only cells with proliferative ability, it is possible to selectively kill them through whole-body X-ray irradiation [71], [72]. Intact planarians were placed on wet filter paper on ice and lethally irradiated at the following conditions: 100 Gy for *S. mediterranea* [15] and 30 Gy for *D. japonica* [68] using an IBL 437C gamma-irradiator (CisBioInternational), 59 Gy/min. Some intact *D. japonica* specimens were exposed to a sublethal dose (5 Gy) of X-Ray (200 KeV, 1 Gy/min) [73], using a Stabilipan 250/1 instrument (Siemens, Gorla-Siama, Milan, Italy) equipped with a Radiation Monitor 9010 dosimeter (Radcal Corporation, Monrovia, CA, USA). Irradiated animals were then sacrificed after 2, 4, 7 days for subsequent experiments.

### *In Situ* Hybridization

Whole mount *in situ* hybridization was performed in intact and regenerating animals according to [74] with minor modifications [75]. All digoxigenin-labelled riboprobes were prepared using digoxigenin-labeling kit (Roche), according to the manufacturer’s instructions. Color development of the alkaline phosphate-coniugated anti-DIG antibody was carried out with a mixture of BCIP/NBT (Sigma).

### TUNEL Assay on Cryosections

A TUNEL apoptosis detection kit (ApopTag Red In situ Apoptosis Detection Kit, Chemicon International) was used for DNA fragmentation fluorescence staining. Briefly, animals were killed in 2% HCl/Holtfreter 5/8, fixed in 4% paraformaldehyde for 2 hours, cryoprotected with 20% sucrose and included in Tissue*-*tek (Tissue Freezing Medium, Durham, NC, USA). Cryosections (16 µm) were prepared using a Leica CM1850 cryostat. After 2 hours at room temperature the sections were frozen at −70°C and incubated with a reaction mix containing biotin-dUTP and terminal deoxynucleotidyl transferase for 60 min. Positively stained cells were visualized with fluorescence microscopy and quantified.

### H3P Staining and Immunofluorescence

Immunostaining was performed according to [76] with anti-Synapsin (anti-SYNORF1, Developmental Studies Hybridoma Bank) 1∶50 and anti-arrestin VC-1 (a kind gift from H. Orii) 1∶2000. Alexa488 or Rodamine Red-X (Molecular Probes) 1∶1000 were used as secondary antibodies. The mitotic cells (H3P positive) were detected with α-phosphorylated histone H3 (Upstate) 1∶500, and counted and normalized as described by [26].

### RNAi Experiments

Double-stranded RNA (dsRNA) was synthesized according to [77]. Intact *S. mediterranea* specimens were ventrally injected for 5 consecutive days with 3×32 nl of 1 µg/µl *Smed-mmp1* or *Smed-mmp2-*dsRNA. Intact *D. japonica* specimens were also injected with *Dj-mmp1-* or *Dj-mmp2-*dsRNA using the same protocol. Some planarians were then amputated and allowed to regenerate. The fragments were then re-injected on alternate days and regeneration was monitored daily. Co-injection of *mmp1-* and *mmp2-*dsRNA in both species did not generate synergism or different phenotypes with respect to those observed after individual *mmp1*-dsRNA injections. *Smed-mt-mmpA(RNAi)* and *Dj-mt-mmpA(RNAi)* was carried out on *S. mediterranea* and *D. japonica*, respectively. *Smed-mt-mmpB(RNAi)* was performed on *S. mediterranea.* Microinjection schedule for *Smed-mt-mmpA(RNAi)* and *Smed-mt-mmpB(RNAi)* consisted in daily injections administered for 5 consecutive days with 3×32 nl of 1 µg/µl dsRNA. Some specimens were then amputated. Both intact animals and regenerating fragments were re-injected for 3 times on alternate days. The newly formed blastema of the regenerating fragments was again amputated after 11 days from the first injection and the phenotypes were evaluated daily under the microscope at the second round of regeneration. No synergism or different phenotypes with respect to *Smed-mt-mmpA(RNAi)* were obtained by the co-injection of *Smed-mt-mmpA*-dsRNA and *Smed-mt-mmpB*-dsRNA. The same protocol was used for *Dj-mt-mmpA*-dsRNA injections in *D. japonica*. Planarians injected with *βeta-galactosidase (β-gal)* dsRNA or water were used as negative controls. The efficiency and specificity of each RNAi treatment was checked (Figure S1). The target areas used for RNAi experiments in *S. mediterranea* are: *Smed-mmp1* 13 to 632 bp and 632 to 1517 bp, *Smed-mmp2* 75 to 746 and 589 to 1040 bp, *Smed-mt-mmpA* 45 to 500 bp and 1114 to 1676 bp, *Smed-mt-mmpB* 937 to 1353 bp and 1331 to 1944 bp.

### Real Time RT-PCR

Total RNA was extracted with NucleoSpin RNAII kit (Machery-Nagel). SYBR Green chemistry-based RT-PCR (GoTaq 2-Step RT-qPCR System, Promega) was carried out on a Rotor-Gene 6000 Real time-PCR (Corbett Research). Real Time RT-PCR was performed at least three times with independent RNA samples, according to the experimental design described by [78]. To analyze gene expression stability, Ct values of two *D. japonica* reference genes (*D. japonica Elongation factor-2* and *D. japonica beta actin*) and three *S. mediterranea* reference genes (*S. mediterranea Elongation factor-2*, *S. mediterranea Glyceraldehyde 3-phosphate dehydrogenase* and *S. mediterranea ribosomal protein L13*) were evaluated using Normfinder stability software (Version 0.953). Under our experimental conditions, the most stable reference genes were *Elongation factor-2* for *D. japonica* and *Glyceraldehyde 3-phosphate dehydrogenase* for *S. mediterranea.* The sequences of primers are available upon request.

### BrdU Detection

BrdU detection was performed according to [79]. The number of BrdU positive cells was calculated after 48 h from microinjection of 10 mg/ml BrdU freshly prepared (Sigma) and represents the total count of positive cells anterior to the photoreceptors in a pool of worms (n = 15).

### Statistics and Image Collection

Data tabulation and descriptive statistics were performed with Microsoft Excel. The statistical analysis was performed by means of One-way analysis of variance (ANOVA) or unpaired Student’s t-test. According to Shapiro-Wilk test, the distribution of the logarithmically transformed data was not significantly different from that of a normal distribution. In addition, homoscedasticity analysis, determined with Hartley’s F max test, revealed no differences in variability between the groups (in all samples the F max calculated was lower than the critical value at 0.05).

NBT/BCIP-developed whole mount images were captured on a Nikon SMZ1500 stereomicroscope with CoolSNAP cf – Photometrics camera and NIS-Elements Microscope Imaging Software. NBT/BCIP wax section images were captured using Nikon Eclipse E600 microscope with Digital Sight Camera NIS-Elements Microscope Imaging Software. BrdU signal was analyzed using a Nikon Eclipse Ti Inverted Microscope with Digital Sight Camera and NIS-Elements Microscope Imaging Software. BrdU signals (in green) were superposed upon the bright-field images (in brown). Adobe Photoshop was used to orient, scale and adjust images and improve clarity. Data were neither added nor subtracted; original images are available upon request.

### Analysis of Caspase-3 Activity

Experiments were performed in *Smed-mmp1(RNAi)* animals after 4 days of injection and in a *β-gal (RNAi)* controls, in triplicate. The animals were washed in cold calcium, magnesium-free medium (CMF) and then chopped into small pieces, transferred into 250 µl of CMF plus papain (30 U/ml, Sigma) for 1 h at 25°C. The lysates were filtered in a Nitex membrane (pore diameter 150 µm) and then added to the lysis buffer (Tris-HCl 20 mM pH 7.4, 150 mM NaCl, 1 mM DTT, 5 mM EDTA, 5 mM EGTA, 0,1% TRITON X-100). Lysates were cleared by centrifugation at 1000 rpm for 15 min at 4°C. Protein content was determined by Bradford method, using the Bio-Rad protein assay kit (Bio-Rad Laboratories) and bovine serum albumin as a standard. Determination of Caspase-3 activity was carried out in a 96-well plate in a total volume of 50 µl. Extracts were incubated for 4 h in the presence of the corresponding tetrapeptide conjugated to paranitroaniline (pNA) and the released pNA was measured in a spectrophotometer (Ultra Microplate reader Bio-Tek Instruments Inc) at 405 nm every 30 min using KC Junior software. The values of *Smed-mmp1(RNAi)* samples were normalized with respect to the controls, after 2 h of reaction.

### Transmission Electron Microscopy

*Dj-mt-mmpA(RNAi)* planarians were fixed in 25% (v/v) glutaraldehyde in 0.1 M cacodylate buffer and post-fixed with 2% osmium tetroxide. Ultrathin sections were stained with uranyl acetate and lead citrate, and observed with a TEM JEOL 1010 (30KVolts) transmission electron microscope. The pictures were captured with a mega view camera using a soft analysis system.

## Results

### Structure and Genomic Organization of Planarian *mmp*-related Genes

**Figure 1:**
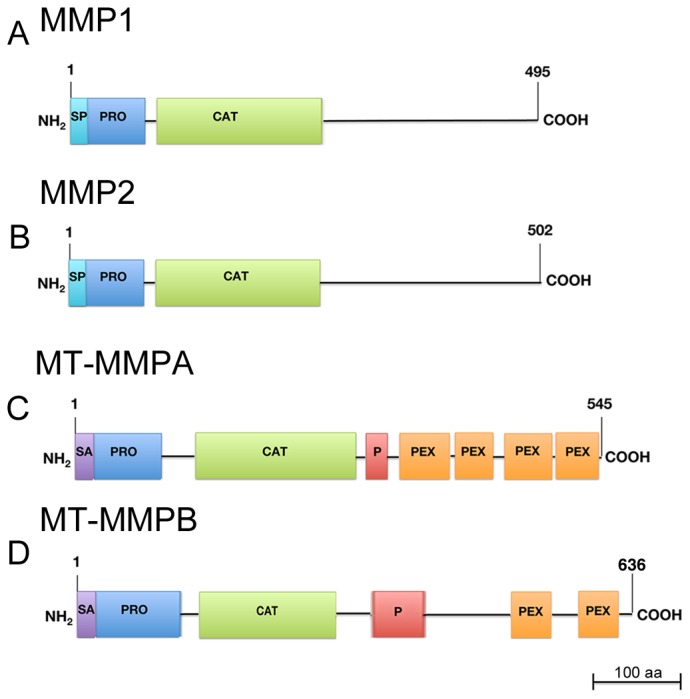
Schematic of the structural organization of the predicted planarian MMPs. (A) MMP1. (B) MMP2. (C) MT-MMPA. (D) MT-MMPB. As deduced by the analysis in *S. mediterranea*, planarian *mmp1* and *mmp2* encode minimal MMPs, consisting of the pro-domain (including the signal peptide) and the catalytic domain (A and B). Planarian *mt-mmpA* and *Smed-mt-mmpB* encode proteases containing a pro-domain and the catalytic domain, connected by a proline-rich hinge to 4 and 2 hemopexin repeats, respectively. The presence of a putative N-terminal anchor signal indicates that these proteins may be membrane-tethered (C and D). SP: signal peptide. PRO: prodomain. CAT: catalytic domain. SA: anchor signal. P: proline-rich hinge. PEX: hemopexin-like domain.

In this paper we focus on the characterization of four planarian *mmp*-related genes, identified by *in silic*o analysis in *S. mediterranea.* Two of them, *Smed-mmp1* and *Smed-mmp2*, encode putative matrilysins (minimal MMPs) [80]. SMED-MMP2 shares 44% identity with SMED-MMP1. The other genes (*Smed-mt-mmpA* and *Smed-mt-mmpB*) encode putative proteases, which are very different from SMED-MMP1 and SMED-MMP2 (Figure 1; Figure S2 **)**. Based on the structural characteristics, MT-MMPA and SMED-MT-MMPB can be considered *bona fide* transmembrane MMPs with Ncyto/Cexo topology, similar to type II MT-MMPs [81]. All planarian MMPs are characterized by a pro-domain and a zinc-binding motif with potential catalytic properties (Figure 1A–D; Figure S2). Both SMED-MT-MMPA and SMED-MT-MMPB contain an additional proline-rich hinge region, hemopexin-like motifs and a potential membrane anchor domain at the NH2 terminus. These two proteins are very different from each other (21% identity). Four hemopexin-like motifs typify SMED-MT-MMPA, while only two can be predicted by SMART in SMED-MT-MMPB. A furin cleavage site has been identified in SMED-MT-MMPA, but not in SMED-MT-MMPB. Finally, SMED-MT-MMPB has unusual repeats TTTPEP in its hinge domain (Figure 1C, D; Figure S2). Comparison of the genomic organization shows that *Smed-mmp1* and *Smed-mmp2* have very similar intron/exon organization, with ten exons of comparable size. *Smed-mt-mmpA* and *Smed-mt-mmpB* consist of nine exons, but the sizes and organization differ between the two genes (Figure S3). To illuminate the evolutionary history of the planarian MMPs, we performed a phylogenetic analysis. We recover most of the MMP sub-families into separate clusters; some of the mismatched sequences had only homology evidences on the databases, which can point out errors in the assignment of those sequences to a given sub-family. We also observe a good correspondence with the domain structures along the clusters. The bootstrap values are higher on vertebrate clusters than for the rest, mainly due to the composition of the NCBI-Homologene sequence sets. However, many of the internal nodes have low bootstrap scores, also due to the lack of a larger set of homologous proteins for species coming from a more diverse background of taxonomic groups, for instance we lack representatives of lophotrocozoa groups in many MMP clusters (with some exceptions like in MMP19 and MMP24 sub-families). The phylogenetic tree shows that SMED-MT-MMPB and SMED-MT-MMPA are somehow close to human MMP19 and MMP25, respectively. SMED-MMP1 and SMED-MMP2 are closer to the SMED-MT-MMPB than to any other sequence, suggesting an expansion of this family in *S. mediterranea*. SMED-MMP1 and SMED-MMP2 seem to be the result of a more recent duplication. Despite the phylogenetic analysis does not provide enough support for such assertion, as sequences are quite divergent, domain analysis using HMM profiles yields further evidences for their relatedness (Figure S3).

An *in silico* search and PCR-based approaches identified the *mmp* counterparts in the related planarian *D. japonica.* We succeeded in characterizing these genes that appeared almost identical to those found in *S. mediterranea* (Figure S2).

### *mmp1* and *mmp2* are Expressed in a Subset of Secretory Cells in *S. mediterranea* and *D. japonica*

**Figure 2:**
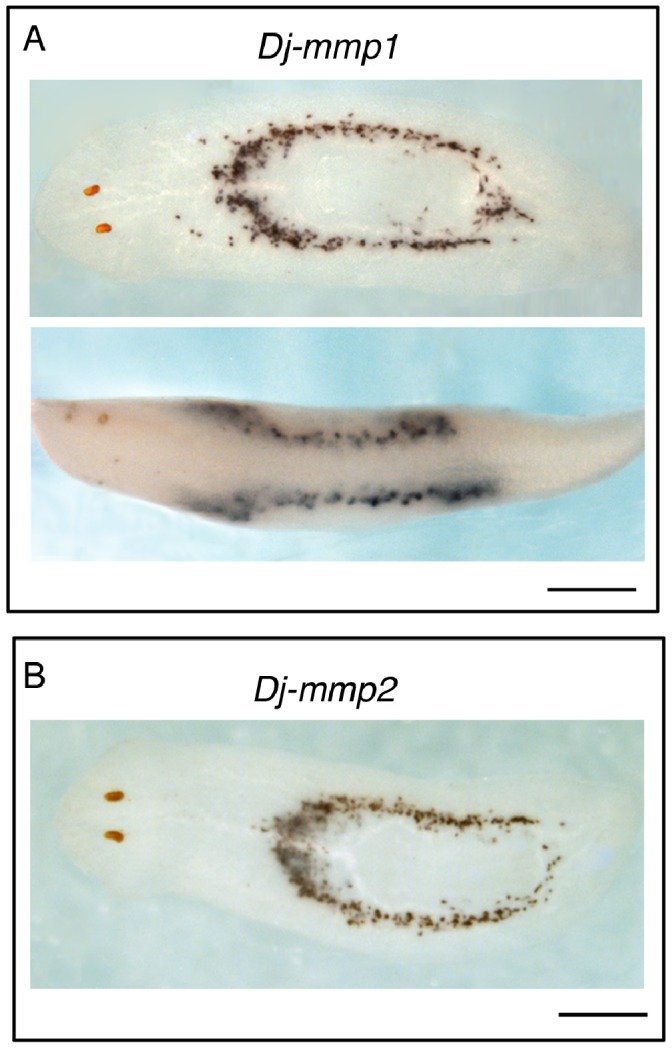
Expression pattern of *mmp1* and *mmp2* in *D. japonica*. (A) Dorsal and lateral view of an intact planarian after WISH shows distribution of *Dj-mmp1* expression in two ring-shaped regions, localized dorsally and ventrally in the central region of the body. (B) Dorsal view of an intact planarian depicting the expression of *Dj-mmp2* after WISH. Anterior to the left. Scale bars: 1 mm.

The spatial expression of the planarian *mmp* genes has been characterized by whole mount *in situ* hybridization (WISH) both in *S. mediterranea* and *D. japonica*. In intact animals *Smed-mmp1* and *Smed-mmp2*, as well as *Dj-mmp1* and *Dj-mmp2*, are strongly expressed in large cyanophilic secretory cells confined to the central body region, in two ring-shaped areas localized dorsally and ventrally in the parenchyma around the pharynx (Figure 2A, B; Figure S4). After wounding, expression of both genes did not change and remained localized in two ring-shaped areas during regeneration of trunk fragments. Some labeled cells were again detected in head and tail fragments only after 4–5 days of regeneration. Then the typical pattern reconstituted (Figure S4).

### *mt-mmpA* and *mt-mmpB* Transcripts are Localized in Postmitotic Cells Widely Distributed in the Planarian Body

**Figure 3:**
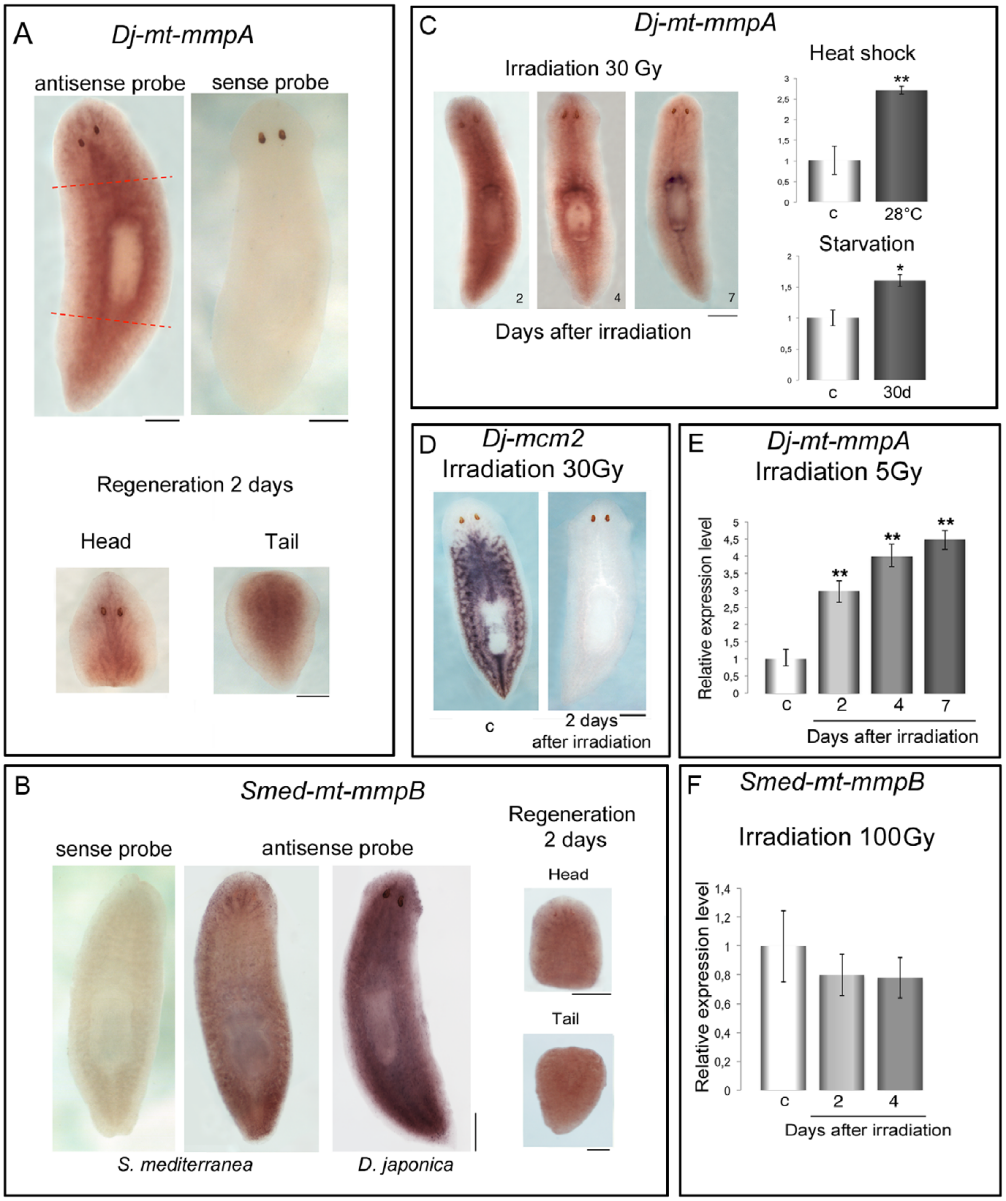
Expression of *mt-mmpA* in *D. japonica and mt-mmpB in S. mediterranea*. (A) WISH with antisense *Dj-mt-mmpA* probe in an intact planarian shows widespread distribution of *Dj-mt-mmpA* expression. Absence of hybridization signal with sense *Dj-mt-mmpA* probe demonstrates signal specificity. *Dj-mt-mmpA* expression pattern in head and tail fragments after 2 days of regeneration is shown. The dotted red lines indicate the regions of the cut that produced the head and tail fragments, respectively. (B) WISH with antisense *Smed-mt-mmpB* probe in an intact planarian shows widespread distribution of *Smed -mt-mmpB* transcripts. Absence of hybridization signal with sense *Smed-mt-mmpB* probe demonstrates signal specificity. Cross hybridization of *Smed-mt-mmpB* riboprobe in *D. japonica* confirms the high level of identity of this gene. *Smed-mt-mmpB* expression pattern in head and tail fragments after 2 days of regeneration is shown. (C) WISH of *Dj-mt-mmpA* in lethally irradiated planarians, sacrificed at different days after irradiation (30 Gy). The effects of heat shock and starvation are also shown. 30d: 30 days. (D) Expression of the stem cell marker *Dj-mcm2* is analyzed by WISH to test the effectiveness of irradiation. c: non-irradiated planarians. (E) Real time RT-PCR analysis of *Dj-mt-mmpA* expression level in planarians irradiated with a sublethal dose of X-rays (5 Gy), and sacrificed at different days after treatment. (F) Real time RT-PCR analysis of *Smed-mt-mmpB* expression level in planarians irradiated with a lethal dose of X-rays (100 Gy), and sacrificed at different days after treatment. The expression level is indicated in relative units, assuming the value of the non-irradiated planarians as unitary. Each value is the mean ± s.d. of three independent samples, analyzed in duplicate. Significant differences in the expression level between irradiated and non-irradiated planarians were detected using the analysis of variance (ANOVA) **P = 0.001. c: non-irradiated planarians. Scale bars: 1 mm.

*Smed-mt-mmpA* and *Dj-mt-mmpA* share a related expression pattern (Figure 3). Both genes exhibit in fact diffuse expression throughout the parenchyma of intact animals, including postmitotic areas. Some cell clusters can be distinguished around the cephalic ganglia and at the epithelial level. *Smed-mt-mmpB* transcripts also show a similar widespread distribution and this expression is conserved in *D. japonica* (Figure 3B). WISH experiments with sense probe, as well as following RNAi, confirmed the specificity of the expression of these genes (Figure 3A, B; Figure S1; Figure S5). Expression pattern of the planarian *mt-mmp*-related genes did not change during regeneration (Figure 3A, B). Because the spatial signal distribution did not allow us to define the cell types expressing *Smed-mt-mmpA*, *Dj-mt-mmpA* or *Smed-mt-mmpB*, we exposed planarians of both species to X-rays to specifically eliminate neoblasts and their progeny, leaving postmitotic cells unaffected. Following irradiation, expression of these genes remained almost constant for at least 4 days post irradiation, while the expression of the neoblast marker *mcm2* appeared markedly downregulated after 2 days (Figure 3C, D, F; Figure S5). These experiments suggest that the planarian *mt-mmp*-related genes are expressed in irradiation-insensitive parenchymal cells. Interestingly, when animals were irradiated with a sub-lethal dose (5 Gy) of X-rays, a significant increase of the expression level of *Dj-mt-mmpA* was observed at 2, 4 and 7 days after exposure (Figure 3E) [73]. Transcriptional induction of *Dj-mt-mmpA* is also detected after heat shock or starvation (Figure 3 C), suggesting that the planarian *mt-mmpA* gene is sensitive at the transcriptional level to harmful situations.

### RNAi of *mmp*1 Causes Tissue Disorganization and Decreased Cell Death during Tissue Homeostasis, but does not Affect Regeneration in *S. mediterranea* and *D. japonica*

**Figure 4:**
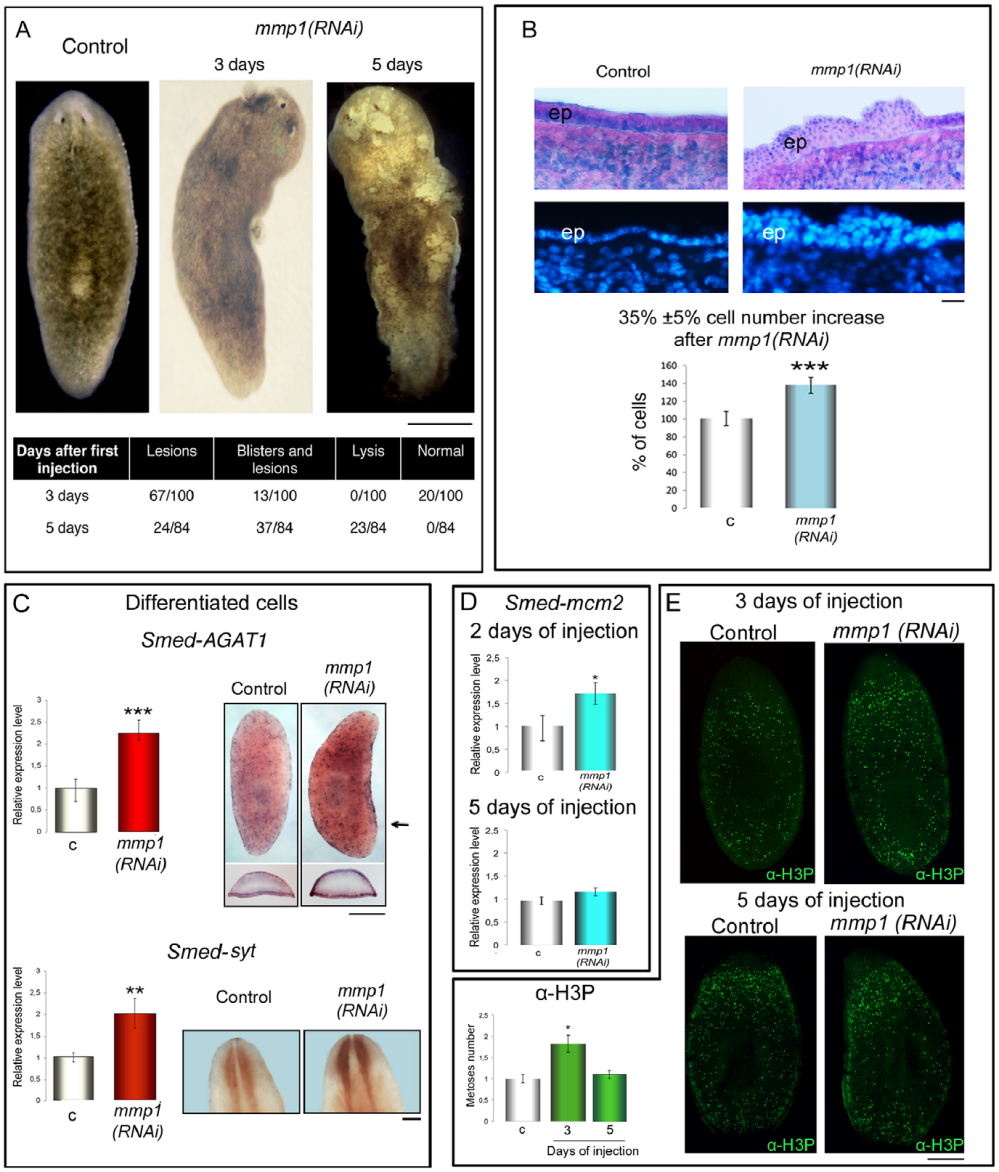
Analysis of *mmp1(RNAi)* phenotype in *S. mediterranea*. (A) Representative images of planarians, as visualized after 3 days and 5 days of injection with *Smed-mmp1* dsRNA. Control represents a planarian at 5 days after *β-gal* dsRNA injection. Formation of lesions and blisters was observed throughout the body of *Smed-mmp1(RNAi)* animals. Quantification of the phenotypes obtained is shown. Scale bar: 1 mm. (B) Longitudinal cryosections stained with hematoxylin/eosin showing similar anatomical areas and illustrating dorsal epithelium (ep) at the prepharyngeal level. c*: β-gal(RNAi)* control; *mmp1(RNAi)*: *Smed-mmp1(RNAi)*. Tissue disorganization with an increase of cell density, disruption of the basement membrane with some cellular infiltrations in the epithelium were observed in *Smed-mmp1*(*RNAi)* animals. In similar regions nuclei are visualized in blue with Hoechst 33342. Scale bar: 50 µm. The graph shows a significant increase in the number of cells in *mmp1(RNAi)* animals compared to controls. Total cell numbers in controls and *Smed-mmp1(RNAi)* phenotypes were calculated as described by [26]. Results represent average ± s.d. from 10 planarians in each condition assuming as 100% the value of control planarians. (unpaired t-test) ***P<0.0001. (C) Real Time RT-PCR analysis and WISH demonstrate that the expression level of differentiated cell types, such as *Smed-AGAT1* (the arrow indicates the level of the transverse sections shown in the insets) and *Smed-syt,* is significantly increased in *Smed-mmp1(RNAi)* phenotypes with respect to the controls. (D) Real Time RT-PCR analysis of *Smed-mcm2* at 2 days and 5 days of injection with *Smed-mmp1* dsRNA. A significant increase of the expression level between *Smed-mmp1(RNAi)* phenotypes and *β-gal(RNAi)* controls is detected at 2 days of injection. On the contrary, no difference in the expression level between *Smed-mmp1(RNAi)* phenotypes and controls is detected at 5 days of injection. In the Real Time RT-PCR experiments the expression level is indicated in relative units, assuming as unitary the value of the controls. Each value is the mean ± s.d. of three independent samples, carried out in duplicate. (unpaired t-test) *P<0.05, **P<0.001, ***P<0.0001. c: *β-gal(RNAi)* controls; *mmp1(RNAi)*: *Smed-mmp1(RNAi)*. Scale bars: 500 µm. (E) Representative images of an intact *β-gal(RNAi)* control and *Smed-mmp1(RNAi)* planarian, after labeling with anti-H3P antibody at 3 days and 5 days of injection, respectively. After 3 days of injection H3P-signal reveals an increased mitotic activity in *Smed-mmp1(RNAi)* animals compared to the controls. After 5 days of injection no difference in the H3P-signal is detected between RNAi animals and controls. The number of mitotic cells in both experimental groups, quantified as described [26], is shown in the graph. Values represent average ± s.d. from n = 22 samples assuming as unitary the number of mitoses in control animals. (unpaired t-test) *P<0.05. Scale bar: 1 mm.

To elucidate the function of *mmp*-related genes in planarians, we inhibited their expression with the use of RNAi. *Smed-mmp2*(*RNAi)* or *Dj-mmp2*(*RNAi)* planarians did not show any abnormal phenotype (Figure S1). In contrast, RNAi of *Smed-mmp1* or *Dj-mmp1* resulted in the formation of lesions and blisters covering the dorsal surface, which multiplied after successive injections and were eventually followed by lysis of the animal (Figure 4A). The regeneration process did not appear compromised. All injected fragments healed wounds and were still capable of forming blastema with similar dynamics as control animals (Figure S6). Histological analysis of *Smed-mmp1(RNAi)* intact animals revealed widespread disorganization of tissues, while degradation of the basement membrane induced infiltration of cells, producing multiple cell layers in the epithelium. Moreover, a significant increase in cell density was detected when compared with controls. This suggests that relevant alterations in tissue homeostasis occurred as a consequence of *Smed-mmp1* knockdown (Figure 4B). To further investigate this result we characterized the relative expression level of cell-specific molecular markers. Under our experimental conditions (planarians injected on 5 consecutive days with dsRNA *Smed-mmp1*), expression level of *Smed-AGAT1,* a gene marking post-mitotic cell types (category 3: [15]) or *Smed-syt,* specific for nerve cells, was significantly increased (Figure 4C). Conversely, expression the neoblast-specific proliferation marker *Smed-mcm2*, as well as the number of anti-phospho*-*histone H3 (H3P)-positive cells, did not appear affected by *mmp1* silencing at this time (Figure 4D, E). As it is known that disruption of the basement membrane separating epithelial cells and parenchymal tissue is associated with a proliferative response in neoblasts upon amputation [26], [82], [83], [84] we wondered whether basement membrane degradation in *mmp1(RNAi)* phenotypes triggered a transient neoblast hyperproliferation event, that could only be detected in the earliest days of treatment. Both the expression of *Smed-mcm2* and immunostaining with H3P antibody demonstrate that a significant proliferative response is detected in *Smed-mmp1(RNAi)* phenotypes after 2 and 3 days of daily injections (Figure 4D, E).

**Figure 5:**
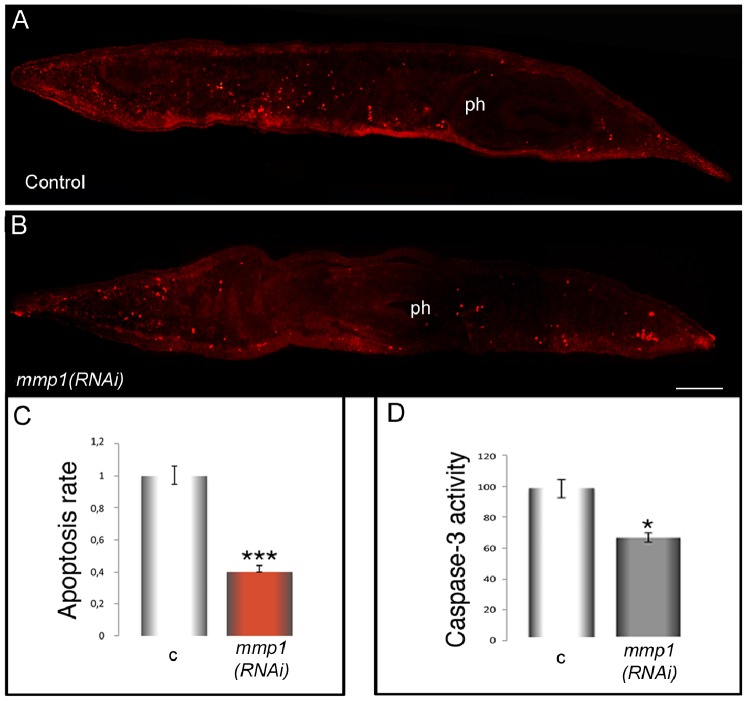
TUNEL assay and caspase-3 activity after *Smed-mmp1(RNAi)* in intact *S. mediterranea*. Representative images of TUNEL-stained longitudinal cryosections from intact planarians. Randomly distributed red spots represent TUNEL-positive nuclei. (A) A *β-gal(RNAi)* control planarian. (B) An *mmp1(RNAi)* planarian exhibits significant diminution of TUNEL-positive spots (approximately 60%) compared to controls, indicating that the number of apoptotic cells decreased as a consequence of *mmp1* knockdown. Scale bar: 1 mm. Anterior to the left. ph: pharynx. (C) The graph represents average ± s.d. of apoptosis rate from the analysis of cryosections obtained from 15 animals, assuming as unitary the value of the control. (D) Caspase-3 activity was determined using the caspase substrate DEVD-pNA (see Methods). c: *β-gal(RNAi)* controls; *mmp1(RNAi)*: *Smed-mmp1(RNAi)*. Data are expressed as percentage and are the mean ± s.d. of 2 independent experiments, performed in triplicate. (unpaired t-test) *P<0.05; ***P<0.0001.

Under normal conditions a precise balance between cell proliferation and apoptosis is required for proper tissue remodeling [21]. To assess whether the unregulated accumulation of differentiated cell types throughout the entire body (as detected at day 5 post-*mmp1(RNAi)*) reflected decreased cell death, we carried out the TUNEL assay. The amount of apoptosis, measured by TUNEL positive nuclei, demonstrates a substantial decrease in the number of apoptotic cells throughout the entire body in *Smed-mmp1(RNAi*) phenotypes compared to control planarians (Figure 5A–C). Similar results were obtained in *D. japonica* following *Dj-mmp1(RNAi*) (Figure S7). We also found significant reduction of caspase-3 activity (approximately 40%) in *Smed-mmp1(RNAi)* animals compared with controls (Figure 5D).

### Silencing of *mt-mmpA* Interferes with Cell Migration during Tissue Homeostasis and Regeneration in *S. mediterranea* and *D. japonica*

**Figure 6:**
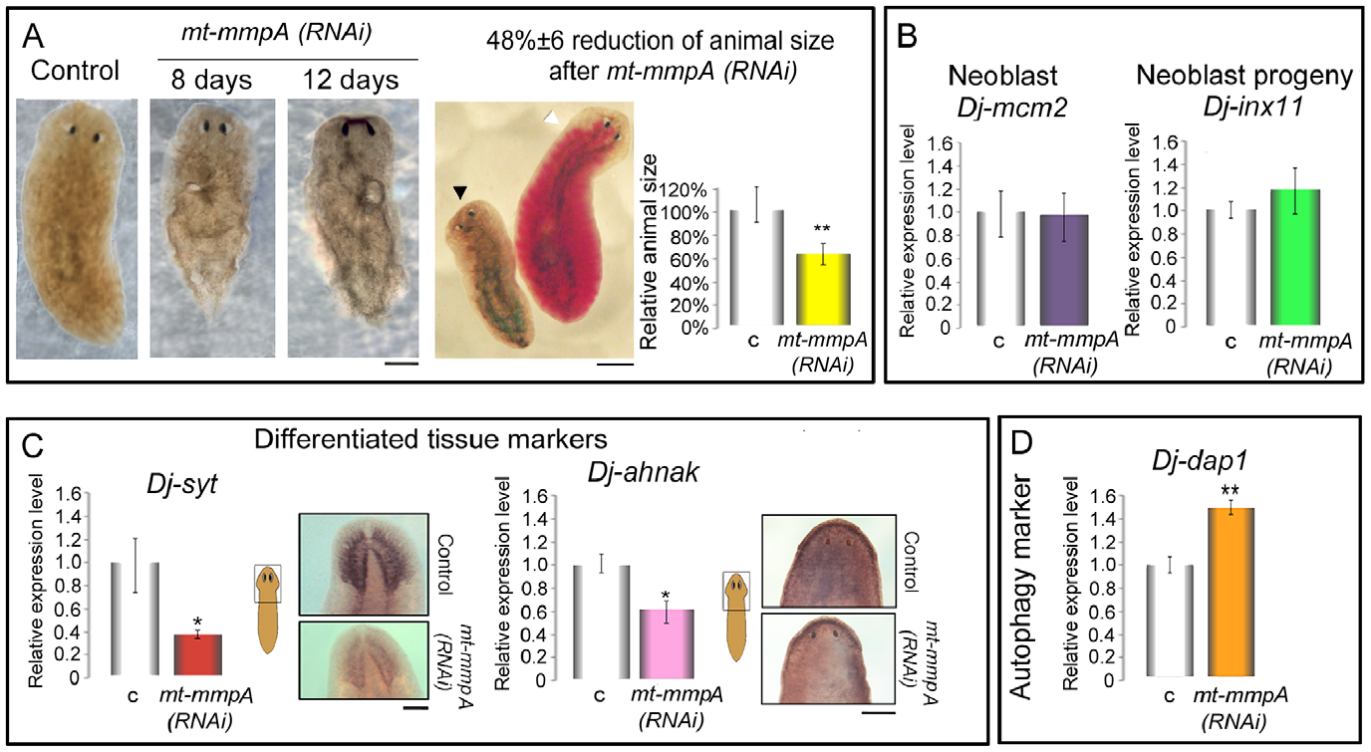
Characterization of *Dj-mt-mmpA(RNAi)* phenotypes in intact *D. japonica*. (A) Bright-field image of a *β-gal(RNAi)* control, at 12 days after the first injection and *Dj-mt-mmpA(RNAi)* planarians at 8 and 12 days after the first injection, respectively. In order to visualize food ingestion, some experimental planarians were fed with chick liver mixed with red food dye after 8 days from the first injection. A *Dj-mt-mmpA(RNAi*) phenotype is visualized on the left (black arrowhead). Green signal in the posterior body part represents residual fast green stain we used to visualize injection procedure. Only control planarians were able to eat, and their intestine appeared red (white arrowhead). All planarians are dorsal view, anterior is up. Scale bars: 1 mm. The graph shows quantification of the body size between *Dj-mt-mmpA(RNAi)* planarians and controls. Values are average ± s.d. from 20 samples. (unpaired t-test) **P<0.001. (B) Real Time RT-PCR analysis reveals that the expression level of *Dj-mcm2* and *Dj-inx11* is not affected by *Dj-mt-mmpA(RNAi)*. (C) A significant decrease in the expression level of differentiated tissue markers (*Dj-syt* and *Dj-ahnak*) has been observed between *Dj-mt-mmpA(RNAi)* planarians and controls by Real Time RT-PCR and WISH. (D) Activation of *Dj-dap1* expression is observed in *Dj-mt-mmpA(RNAi)*-injected animals compared to controls. In the Real Time RT-PCR experiments the expression level is indicated in relative units, assuming as unitary the value of the controls. Each value is the mean ± s.d. of three independent samples, carried out in duplicate. Samples were compared using the unpaired t-test. *P<0.05, **P<0.001. c: *β-gal(RNAi)* control; *mt-mmpA(RNAi)*: *Dj-mt-mmpA(RNAi)*.

Next, we functionally characterized the other planarian *mmp*-related genes, *Smed-mt-mmpA, Dj-mt-mmpA,* as well as *Smed-mt-mmpB*. While planarians injected with *Smed-mt-mmpB* dsRNA were indistinguishable from the controls (Figure S1 and Figure S8), loss of function *of mt-mmpA* caused abnormalities in both *S. mediterranea* and *D. japonica*. *mt-mmpA(RNAi)* specimens of both species developed abnormally soft bodies and multiple lesions and also showed progressive tissue regression in front of the eyes. The results for *D. japonica* are shown in Figure 6A. All *mt-mmpA(RNAi)* animals died within 20 days, while survival of controls was not affected. We also observed that *Dj-mt-mmpA(RNAi)* planarians shrank in size relative to controls, and were unable to eat (Figure 6A). To investigate the effects induced by *mt-mmpA* silencing at the molecular level, we analyzed different markers both in *D. japonica* and in *S. mediterranea*. The expression level of *Dj-mcm2* [85] and *Dj-inx-11* [75], as well as that of *Smedwi-1* [15] and *Smed-prog-1* (*Smed-NB.21.11e*: [15]) did not change significantly in intact animals following *mt-mmpA(RNAi)* (Figure 6B; Figure S9). In contrast, the level of expression of markers of differentiated cell types (*Dj-syt* and *Dj-anhak* in *D. japonica*; *Smed-AGAT1* and *Smed-Syt* in *S. mediterranea*) appeared significantly reduced (Figure 6C; Figure S9). As these results imply possible activation of autophagic processes, we also analyzed the expression level of *Dj-dap1* and *Smed-dap1*, homologs of *Gtdap-1* (death-associated protein 1), which is considered a positive mediator of autophagy/autophagic cell death in the planarian *Girardia tigrina* [86]. Our results show a significant increase in the level of *dap1* transcripts in *mmp1(RNAi)* planarians compared to controls (Figure 6D; Figure S9).

**Figure 7:**
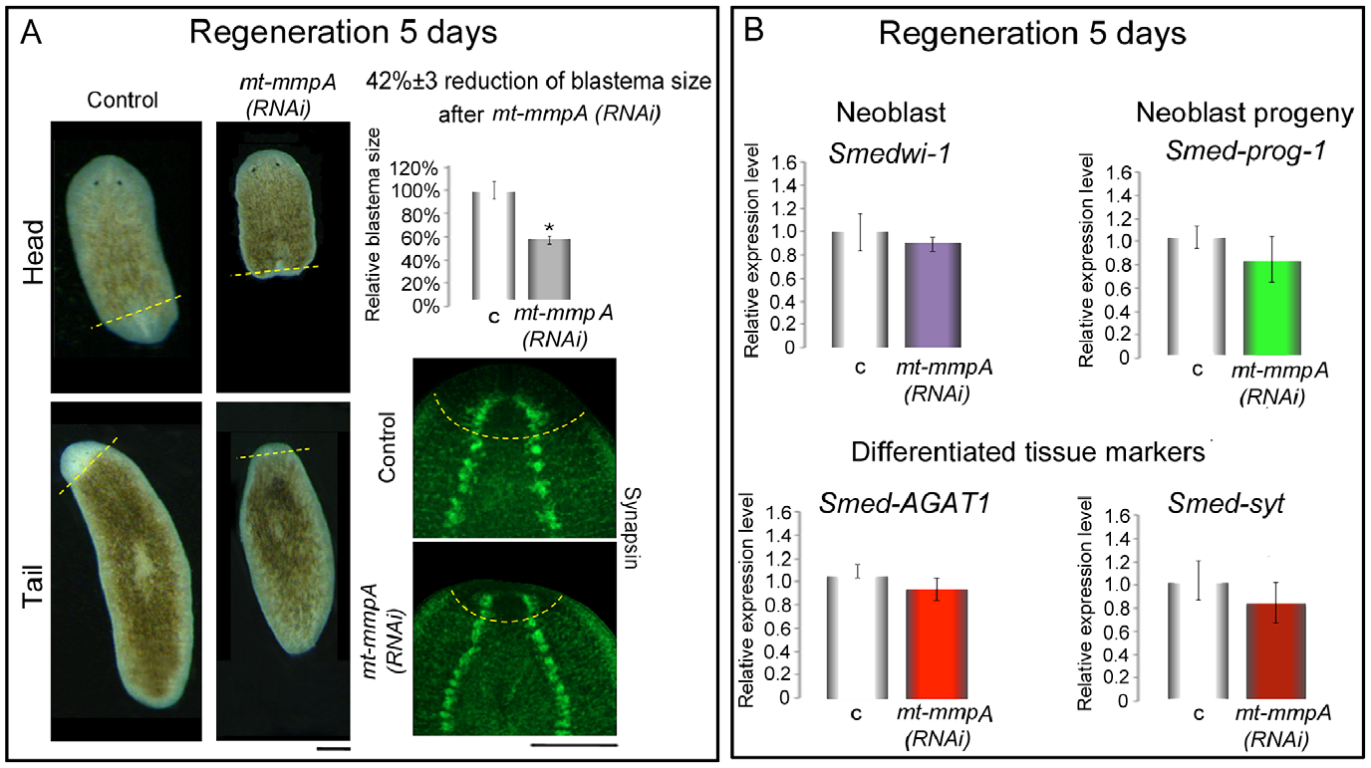
Characterization of *mt-mmpA(RNAi)* phenotypes in regenerating *S. mediterranea*. (A) Bright–field images of head and tail fragments, after two rounds of dsRNA injections and amputation, at 5 days of regeneration. Control: *β-gal(RNAi)* fragments. Following *Smed-mt-mmpA*(*RNAi)* the fragments show a significantly reduced blastema. Relative blastema size between *Smed-mt-mmpA(RNAi)* and *β-gal(RNAi)* fragments was calculated as percentage of the whole fragment, considered 100%. Consistent with the delayed regeneration phenotype detected at the morphological level, immunostaining with anti-synapsin antibody of *Smed-mt-mmpA(RNAi)* tail fragments (green signal) reveals a regenerating brain smaller than that of controls. The dashed yellow lines indicate the border between the regenerating area and the stump. Anterior is up. Scale bars: 1 mm. (B) Expression level of different markers (*Smedwi-1, Smed-prog1*, *Smed-AGAT1*, *Smed-syt*) after *Smed-mt-mmpA(RNAi)*, analyzed by Real Time RT-PCR in regenerating fragments. No significant variation in the expression level of different markers was observed. In the Real Time RT-PCR experiments the expression level is indicated in relative units, assuming as unitary the value of the controls. Each value is the mean ± s.d. of three independent samples, carried out in duplicate. c: *β-gal(RNAi)* controls; *mt-mmpA(RNAi)*: *Smed-mt-mmpA(RNAi)*.

When amputated, *mt-mmpA(RNAi)* specimens of both species regenerated very slowly (132/180, 73% in *D. japonica* and 51/60, 85% in *S. mediterranea)*. An analysis of the blastema area (morphologically identified as unpigmented tissue), expressed as a percentage of overall fragment, provided quantitative evidence that RNAi-mediated downregulation of this gene delayed blastema growth to the correct proportion. This is consistent with our observation that regenerating cephalic ganglia in *Smed-mt-mmpA(RNAi)* fragments are smaller than those in the controls (Figure 7A). Most interestingly, we observed that *Smed-mmpA(RNAi)* did not interfere with the expression of *Smedwi-1*, neither with the expression of *Smed-prog-1*, markers of neoblasts and neoblast progeny, respectively. The expression levels of differentiated tissue markers (*Smed-AGAT1* and *Smed-syt*) were also unaffected (Figure 7B). Similar results were obtained in regenerating *D. japonica.* No significant difference in the expression level of diverse stem cell/progenitors markers (*Dj-mcm2*, *Djinx-11* and *Dj-bruli*) or differentiated cells from different tissues (*Dj-inx1*, *Dj-ahnack*, *Dj-syt*, *Dj-gad*) could be detected after *Dj-mmpA(RNAi)* in fragments that regenerated a small blastema (Figure S10). *Djdap-1* expression level between *mt-mmpA(RNAi)* fragments and controls did not also change (Figure S10).

**Figure 8:**
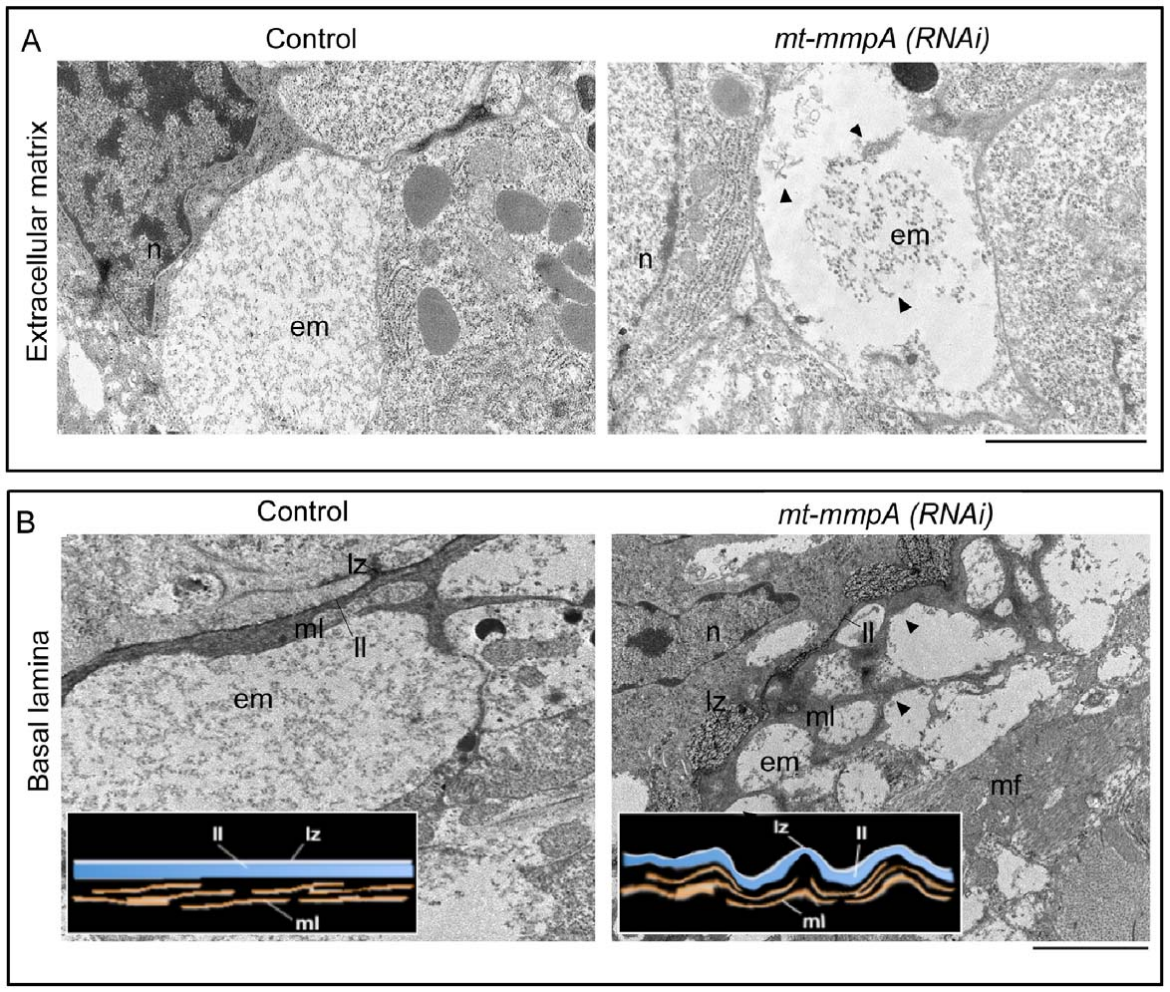
ECM analysis of *Dj-mt-mmpA(RNAi)* in intact *D. japonica* by TEM. (A) Representative micrographs showing a planarian ECM region in a *β-gal(RNAi)* control and following *mt-mmpA(RNAi)*. (B) Representative micrographs showing the basal lamina organization in a *β-gal(RNAi)* control and following *mt-mmpA(RNAi)*. Insets are schematic drawings of the basal lamina structure, as detected in controls and *mt-mmpA(RNAi)* samples, respectively. n: nucleus; em: extracellular matrix; ml: microfibrillar layer; mf: muscle fibres; ll: limiting layer; lz: electron lucent zone. Arrowheads indicate alterations in ECM (A) and basal lamina (B). Scale bars: 2 µm. *mt-mmpA(RNAi)*: *Dj-mt-mmpA(RNAi)*.

To further assess the structural characteristics of *mt-mmpA(RNAi)* intact planarians, we compared RNAi-treated animals with water-injected controls at the ultrastructural level. Transmission electron microscope (TEM) observations, performed in *D. japonica,* show that pericellular ECM in the parenchyma is an electron-dense, finely granular structure (Figure 8) [87]. TEM analysis reveals that the ECM of *Dj-mt-mmpA(RNAi)* phenotypes has a disorganized structure, and most fibers seem aggregated and collapsed on each other (Figure 8A). In addition, the general architecture of the basal lamina - a layer of extracellular matrix anchoring epithelium - became folded upon itself and its components, namely the electron-lucent zone (lz), the limiting layer (ll) and the microfibrillar layer (ml) [83], [84], appeared folded and more disorganized than in controls (Figure 8B). This abnormal organization was mainly detectable in lz and ll, composed essentially by laminin, fibronectin, collagen IV, proteoglycans and integrins [24], [25].

**Figure 9:**
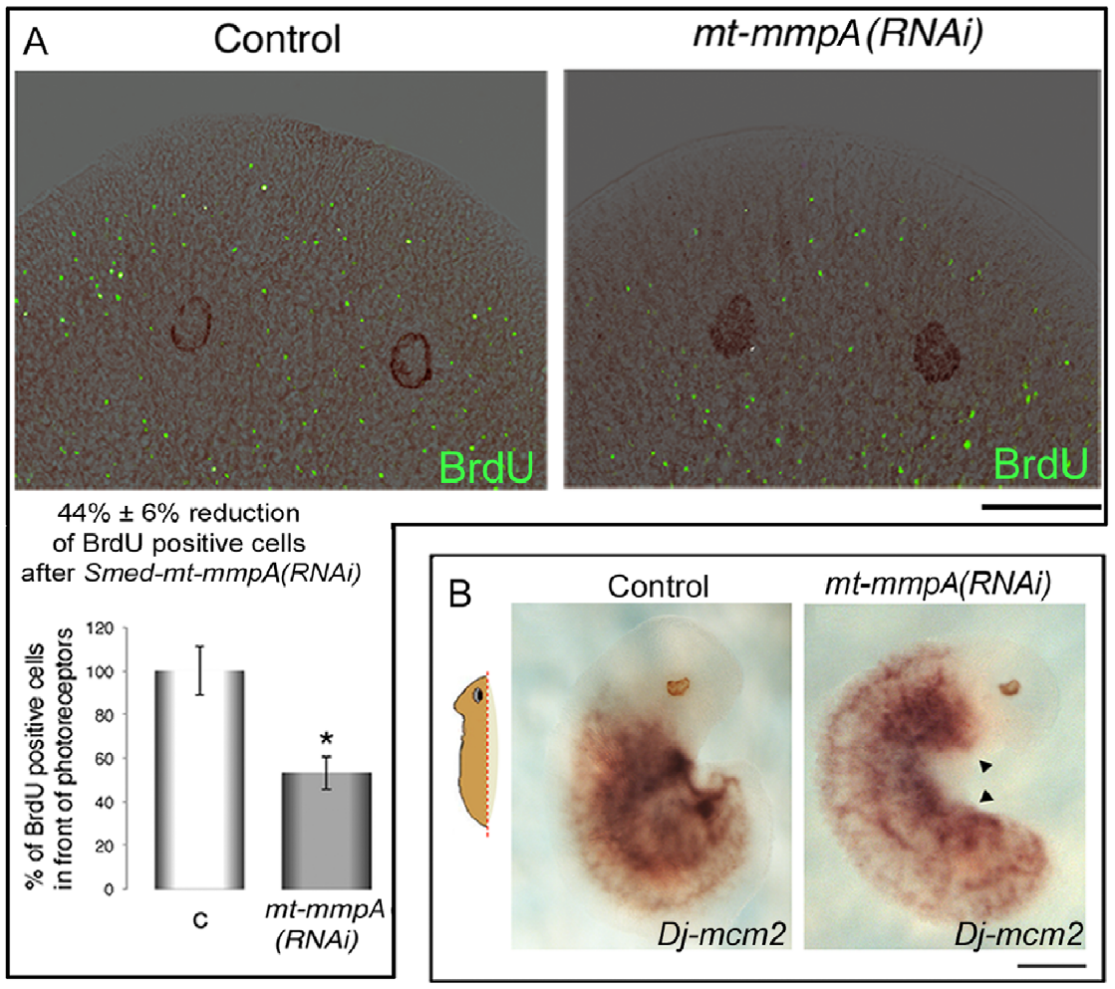
Effect of planarian *mt-mmpA(RNAi)* on cell migration. (A) A representative image of the head region of a *β-gal(RNAi)* control and an *mt-mmp(RNAi)* in *S. mediterranea* is shown. Cell migration is visualized by BrdU labeling (in green) superposed upon a bright-field image (in brown). Both controls and *mt-mmpA(RNAi)* planarians were fixed at 48 h after BrdU injection. At this time numerous cells have moved into the region anterior to the eyes [65]. Anterior is up. Scale bar: 500 µm. The graph shows the significant decrease in the percentage of BrdU-positive cells anterior to eyes in *mt-mmpA(RNAi)* injected animals compared to controls. Results represent average ± s.d. from 15 specimens assuming as 100% the value of control planarians. (unpaired t-test) *P<0.05. *mt-mmpA(RNAi)*: *Smed-mt-mmpA(RNAi)*. (B) *Dj-mcm2* expression in lateral regenerants of *D. japonica*, as visualized by WISH in *β-gal(RNAi)* controls and *mt-mmpA(RNAi*) planarians after two rounds of dsRNA injections and amputation, at 2 days of regeneration. In controls the old tissue regenerates the missing lateral part of the body and *Dj-mcm2-*positive cells redistribute to form a radially shaped hybridization signal orientated towards the blastema [71]. No accumulation of hybridization signal is observed in a comparable fragment following *mt-mmpA(RNAi)* (arrowheads). Anterior is up. Scale bar: 1 mm. *mt-mmpA(RNAi)*: *Dj-mt-mmpA(RNAi)*.

We used BrdU labeling to investigate whether the disorganization of the ECM may counteract cell migration. We compared the area anterior to the photoreceptors of controls and *Smed-mt-mmpA(RNAi)* animals. As this region is devoid of dividing neoblasts, the presence of BrdU-positive cells provides evidence of cell migration during physiological cell turnover [79]. We found that the number of BrdU-positive cells migrating into this area was significantly lower in *Smed-mt-mmpA(RNAi)* animals than in controls, 2 days after BrdU pulse (Figure 9A). Moreover, we observed that, after 6 days of regeneration, a small number of H3-positive stem cells migrating into the blastema could be found in control fragments. On the contrary, H3-positive neoblasts were missing in the small blastema of *Smed-mt-mmpA(RNAi)* phenotypes (Figure S11). We also looked at the spatial distribution of proliferating cells by WISH using *Dj-mcm2* as a probe on fragments amputated sagittally after *Dj-mt-mmpA(RNAi)*. We did not observe any substantial accumulation of *Dj-mcm2* signal at the wound site where loss of tissue induces neoblast migration [19], [85]. In addition, *Dj-mcm2*-positive cells did not redistribute to form the typical radially shaped pattern, orientated towards the wound region (Figure 9B; [81]).

## Discussion

### Characterization of Planarian *mmp* Genes

The experimentally accessible stem cell population and the robust regenerative capabilities of planarians offer an attractive model system to study how modulation of the proteolytic system in the extracellular environment affects cell behavior *in vivo* [10]. The limited number of MMP members identified in *S. mediterranea* (*Smed-mmp1*, *Smed-mmp2*, *Smed-mt-mmpA* and *Smed-mt-mmpB*), as well as the conservation of the counterparts in *D. japonica*, raise the possibility that planarians make use of an extremely simple set of *mmp*-related genes to modulate tissue remodeling, when compared with the highly redundant MMP system operating in vertebrates or other invertebrates [88]. All domains of planarian MMPs appear well conserved. However, these enzymes also contain a number of unique features, whose functional significance remains to be determined. For example, no cysteine arrays or immunoglobulin-like domains can be found at the C-terminal of SMED-MT-MMPA and SMED-MT-MMPB, while the presence of hemopexin repeats in this region makes these proteins more similar to vertebrate gelatinases [89]. Moreover, planarian MMP1 and MMP2 show an unusual long C-terminus tail that distinguishes them from all the remaining MMPs described in any organism. Although no annotated domains could be identified in this region, it is suggestive to hypothesize that the long tail can contain motifs recognized by specific planarian inhibitors/modulators, in order to temporally and spatially regulate the proteolytic activity and/or substrate specificity of these proteins.

The structural differences observed between the minimal MMPs and the membrane-type MMPs of planarians can be extended to their respective expression patterns, and are consistent with the possibility that these enzymes play distinct roles in tissue remodeling. Both *Smed-mmp*1 and *Smed-mmp2* in *S. mediterranea* and *Dj-mmp*1 and *Dj-mmp2* in *D. japonica* are in fact strongly expressed by large secretory cells, localized in a discrete region of the parenchyma. Conversely, *Smed-mt-mmpA* and *Smed-mt-mmpB* are expressed in postmitotic cells that are widely distributed throughout the planarian body. A similar pattern is also conserved in *D. japonica*, as *Dj-mt-mmpA* WISH results demonstrate. In other organisms the levels of MMP transcripts are usually low in normal tissues, being their expression rapidly upregulated when active physiological or pathological tissue remodeling takes place [36], [37]. Constitutive production of high levels of MMP transcripts may represent an adaptive mechanism of these plastic animals. To better understand the functional relevance of each MMP we reduced the expression of each gene by RNAi.

### Planarian *mmp1* is Required to Modulate Tissue Homeostasis and Survival of Postmitotic Cells

RNAi of *Smed-mmp-1* or *Dj-mmp-1* resulted in the formation of blisters and disruption of tissue architecture in intact worms. Histological analyses of the phenotypes confirmed extensive tissue disorganization, as well as degradation of the basement membrane, indicating that the expression of this gene is essential in determining the homeostatic remodeling of ECM. Surprisingly, following amputation, *mmp-1(RNAi)* fragments of both species exhibited normal wound healing and capability to regenerate a blastema that was indistinguishable from that of the controls. One potential interpretation of these results is that MMP1 - that has a signal peptide and is likely to function extracellularly - may regulate its activity at different levels, a feature common to secreted proteinases [90], [91]. We suggest that MMP1 activity is primarily regulated at the transcription level in intact animals. Regulation of *mmp1* expression might be crucial during homeostasis. In addition, a certain amount of gene product may be stored in a catalytically inactive state (pro-enzyme form or zymogen). Following amputation, MMP1 might rapidly exert its biological function activating its proteolytic function with post-transcriptional processing. Significant activation of zymogen in the rapidly remodeling tissue environment of an amputated worm might provide functional protein sufficient to promote the production of an ECM that is conducive to blastema formation and growth. This conclusion is supported by the finding that, following injury, planarians release a large amount of collagen-degrading MMP, and its release does not require the expression of genes and their translation into proteins [25]. Conversely, no mature collagenase is released by intact planarians [25]. Increase of MMP activity appears also critical for the onset of the first regenerative stages in different regeneration models, from *Hydra* to mammals, and appears to be crucial to avoid the formation of scar tissue [45], [92], [93], [94], [95].

The discovery that silencing of *mmp1* expression leads to degradation of the basement membrane and triggers unregulated tissue invasion in intact animals supports the possibility that this protease plays protective/tumor-suppressive roles. MMP1 also plays a role in innate immunity in *S. mediterranea* [96]. Although MMPs have long been considered tumor-promoting enzymes, there is an increasing amount of literature demonstrating that the expression of certain MMPs provides a paradoxical protective effect in multiple stages of cancer progression [97], [98], [99]. Taken together, our results suggest that the protective function of MMPs evolved early, before the divergence of vertebrates from invertebrates, and was conserved during the complex evolution of the MMP family.

We have also explored the consequences of *mmp1* silencing on the behavior of planarian cells. As a consequence of *Smed-mmp1(RNAi)* or *Dj-mmp1(RNAi)* in intact *S. mediterranea* and *D. japonica*, respectively, we observed an early hyperproliferative response of neoblasts, followed by a generalized increase in the number of multiple differentiated cell types. TUNEL and caspase-3 activity assays demonstrated that apoptosis was significantly reduced in *mmp1(RNAi)* animals, suggesting that MMP1 functions as a positive regulator of apoptosis in planarians. Apoptotic evasion is a common strategy to increase cell number in tumors [100], [101], [102]. Preventing abnormal proliferation might provide anti-tumorigenic protection in these plastic animals. Activity of different MMPs has been seen to affect apoptosis in rapidly remodeling tissues, including the intestine during *Xenopus laevis* metamorphosis [103] and morphogenesis/involution of the mammary gland [104]. It has been demonstrated that in planarians the pro-apoptotic factor BCL2 is required for cell survival, following the intrinsic apoptotic pathway [21]. *Smed-bcl2(RNAi)* animals show defects similar to those observed after *Smed−/Dj-mmp1(RNAi)*. Although we cannot completely exclude that imbalance between the number of neoblasts and differentiated cells leads to anomalous tissue remodeling with subsequent deterioration and lysis of the animals, it is possible that MMP1 may directly affect apoptosis. There is evidence that matrilysins can induce cell death by the extrinsic pathway, activated by cell membrane-bound “death receptors”, such as cytokines of the tumor necrosis factor (TNF) family [105], [106]. Since TNF-related proteins have not yet been characterized in planarians and molecular information about the extrinsic pathway is still elusive in invertebrates [107], [108], [109], we can only speculate that MMP1 plays a role in a hypothetical extrinsic apoptotic pathway.

### Activity of Planarian *mt-mmp* Mediates Cell Migration

**Figure 10:**
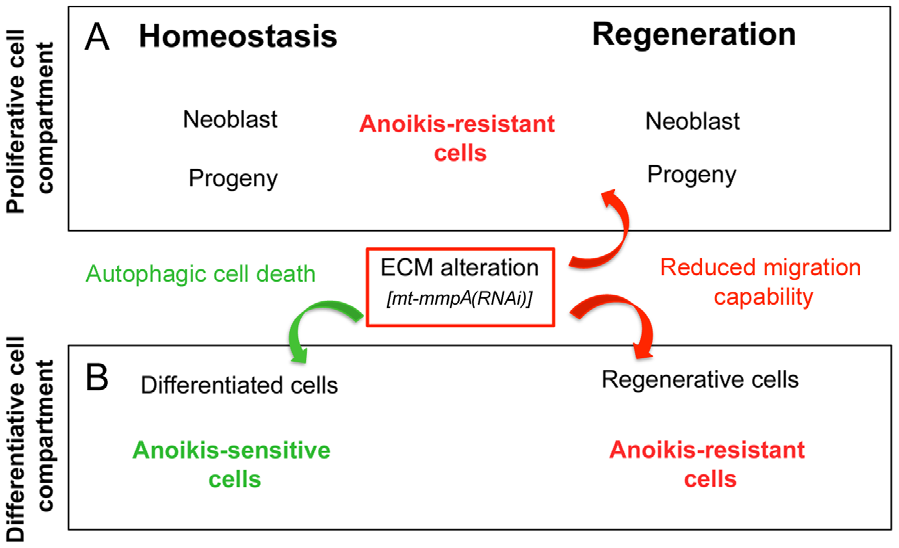
Scheme depicting the physiological relevance of anoikis in relation to homeostasis and regeneration. (A) Anoikis resistance may ensure anchorage-independent growth and survival of cells endowed with proliferative activity, such as neoblasts and their progeny. Prosurvival signals and suppression of death signals might be involved in anoikis resistance [105]. (B) In homeostatic conditions differentiated cells are probably endowed by default with an anchorage-dependent surveillance system in order to maintain the assigned position/function within a given tissue. Autophagic cell death may be induced to inhibit dysplastic cell growth or to prevent attachment to an inappropriate matrix (anoikis-sensitive cells). The ability of planarians to regenerate depends on the capacity both to mobilize stem cells and to remodel differentiated cell types in the pre-existing tissues [18]. Fate-committed regenerative cells and differentiated cells in the pre-existing tissues might become anoikis-resistant cells as a reflection of changes of the ECM in a permissive, regeneration-competent state.

Homeostasis and regeneration are processes critically dependent on the interaction of cells with the surrounding ECM that ensures their correct migration and differentiation. After RNAi of *mt-mmpA* both *S. mediterranea* and *D. japonica* specimens displayed shrunken bodies with lesions and progressive regression of the head. In addition, the expression level of some markers generally employed to characterize differentiated tissues appeared significantly reduced, while no substantial difference in the expression level of stem cell and progenitor cell markers was detected. In light of these results, we hypothesize that normal cell lineage progression and, consequently, proper tissue remodeling may be impaired in intact planarians by *mt-mmpA* silencing. Because in other organisms some membrane-anchored MMPs appear to be involved in the control of cell migration by direct remodeling of the surrounding matrix [110], [111], [112], [113], we analyzed whether the planarian MT-MMPA plays a role in cell migration. Our experimental data support this possibility. Planarians have an ECM with little obvious structure [87]. We suspect that this simple architecture may be more easily “broken-down” to facilitate stem cell mobilization than a highly organized ECM. After *Dj-mt-mmpA(RNAi*), we observed that this simple organization of the matrix was modified. Most fibers appeared aggregated and collapsed on each other, leaving wide empty spaces therein. This abnormal structure might prevent cell migration. Direct evidence that the planarian MT-MMPA is essential for cell migration during tissue remodeling comes from BrdU experiments. We observed in fact that the migration capability of BrdU-stained cells into the region anterior to the photoreceptors of both *Smed-mt-mmpA(RNAi)* and *Dj-mt-mmpA(RNAi)* intact specimens was severely reduced. Recent data demonstrate that neoblasts and undifferentiated progenitor cells do not undergo substantial migration during tissue homeostasis, while amputation triggers stem cell mobilization to sites of injury during planarian regeneration [114]. We wondered whether, after wounding, neoblast movements could also be hampered by *mt-mmpA(RNAi)*. Neither directional mobilization of stem cells toward the amputation site, nor their substantial accumulation in the postblastema area (i.e. the stump region behind the wound), were observed following *Smed-mt-mmpA(RNAi)* or *Dj-mt-mmpA(RNAi)*. Consistent with this, growth of the regenerative blastema appeared strongly delayed. After wounding, characterization of the same molecular markers as in intact animals, revealed no substantial differences in their expression level between *mt-mmpA(RNAi)* fragments and controls. It is remarkable that perturbation of a single cell surface metalloproteinase has different effects on maintenance of tissue homeostasis and regeneration in planarians. On the other hand, homeostatic cell replacement and regeneration occur in different contexts at the organismal level and are regulated by a complexity of signaling pathways that can act independently on ECM or other signaling molecules. The relationship of MMPs with these pathways is still an open question, even if recent data shed new light on the importance of mobilization of stored matrix-associated molecules to evoke tissue regeneration on response to injury [115]. During physiologic/pathologic events (ranging from growth and development to tumor metastasis), stem cells or neoplastic cells cross tissue barriers, largely comprised of collagen - the major structural component of the ECM - using mobilizing proteolytic enzymes [113], [115], [116]. Numerous studies have highlighted the importance of MT-MMPs function in the mobilization of tumor cells of vertebrate and invertebrate species [117], [118]. Mechanistically, inappropriate cell/ECM interactions and/or loss of cell anchorage may compromise the structure of tissues and provoke preferential activation of autophagic cell death for many types of cells via anchorage-dependent apoptosis (anoikis: [119]). Consistent with this possibility, we suggest that, as in other organisms [120], planarian stem cells develop anoikis-resistance as a critical prerequisite to survive detachment from the ECM, thus maintaining their plasticity in homeostasis and regeneration. In this context, it is possible that MT-MMPA mediates processing of regulatory signals and/or ECM structure, promoting their motility and invasive potential (Figure 10A). On the contrary, anoikis-sensitivity could be relevant to determine the correct localization/function of postmitotic cells in tissues. Fate-committed regenerative cells, as well as postmitotic cells of the pre-existing tissues (such as epithelial or nerve cells), could become anoikis-resistent during regeneration to support correct morphogenesis and morphallactic remodeling [121], [122], [123] (Figure 10B).

On the whole, these findings provide evidence that in planarians the cell behavior is critically dependent on extrinsic regulation induced by their microenvironment. In particular, the definition of the complex scenario that involves ECM in the modulation of stem cell behavior has the potential to provide fundamental information that can be of interest for regenerative medicine and cancer research.

## Supporting Information

Figure S1 **Analysis of efficiency and specificity of each RNAi silencing in** ***S. mediterranea*** **.** (A) *Smed-mmp1* expression level as detected by WISH and Real Time RT-PCR in controls and *Smed-mmp1(RNAi)* demonstrates the efficiency of the treatment. Moreover, *Smed-mmp1(RNAi)* does not affect the expression level of *Smed-mmp2*, *Smed-mt-mmpA* and *Smed-mt-mmpB*. (B) *Smed-mmp2* expression level as detected by WISH and Real Time RT-PCR in controls and *Smed-mmp2(RNAi)* animals. Moreover, *Smed-mmp2(RNAi)* does not affect the expression level of *Smed-mmp1*, *Smed-mt-mmpA* and *Smed-mt-mmpB*. (C) *Smed-mt-mmpA* expression level as detected by WISH and Real Time RT-PCR in controls and *Smed-mt-mmpA(RNAi)* animals. Moreover, *Smed-mt-mmpA(RNAi)* does not affect the expression level of *Smed-mmp1*, *Smed-mmp2* and *Smed-mt-mmpB*. (D) *Smed-mt-mmpB* expression level as detected by WISH and Real Time RT-PCR in controls and *Smed-mt-mmpB(RNAi)* animals. Moreover, *Smed-mt-mmpB(RNAi)* does not affect the expression level of *Smed-mmp1*, *Smed-mmp2* and *Smed-mt-mmpA*. The coinjection of *Smed-mt-mmpA* and *Smed-mt-mmpB* does not affect the expression level both of *Smed-mmp1* and *Smed-mmp2*. In the Real Time RT-PCR experiments the expression level is indicated in relative units, assuming as unitary the value of the controls. Each value is the mean ± s.d. of three independent samples, carried out in duplicate. (unpaired t-test) **P<0.001, ***P<0.0001. c: *β-gal(RNAi)* controls; *mmp1(RNAi)*: *Smed-mmp1(RNAi)*; *mmp2(RNAi)*: *Smed-mmp2(RNAi)*; *mt-mmpA(RNAi)*: *Smed-mt-mmpA(RNAi); mt-mmpB(RNAi)*: *Smed-mt-mmpB(RNAi)*; *mt-mmpA/mt-mmpB(RNAi): Smed-mt-mmpA* and *Smed-mt-mmpB(RNAi)*. Scale bars: 1 mm.
(TIF)

Figure S2 **Amino acid sequence alignment of MMP1, MMP2, MT-MMPA and MT-MMPB from** ***S. mediterranea*** **and** ***D. japonica*** **.** (A) *S. mediterranea* SMED*-*MMP1 and *D. japonica* DJ-MMP1. SMED-MMP1 is 495 amino acids in length. The pro-domain consists of 80 amino acids (1 to 80) and contains the pro-peptide cleavage site in position 18 and an atypical cysteine switch (GRCGGKD, 73 to 80). The catalytic domain consists of 175 amino acids (91 to 266) and contains the conserved HEXXHXXGXXH motif (221 to 231). The typical methionine turn is in position 237. (B) *S. mediterranea* SMED*-*MMP2 and *D. japonica* DJ-MMP2. SMED-MMP2 is 502 amino acids in length. The pro-domain (1 to 79) contains an atypical cysteine switch (SRCGGKD, 72 to 79). The propeptide cleavage site is in position 17. The catalytic domain (90 to 265) includes the HEXXHXXGXXH motif (218 to 228). Methionine turn is in position 236. (C) *S. mediterranea* SMED-MT-MMPA and *D. japonica* DJ-MT-MMPA. SMED-MT-MMPA is 545 amino acids in length. The pro-domain region spreads from amino acid 1 to 93. The cystein-switch is detected in position 87 to 93. The presence of a membrane anchor domain (1 to 21) and a furin cleavage site (ILKRVKR: 114 to 120) has been predicted. The catalytic domain consists of 169 amino acids (126 to 295) and contains the conserved motif required for the catalytic activity. Downstream this domain (249 to 259) is a flexible proline-rich hinge region (303 to 326) followed by four hemopexin-like motifs (338 to 389; 394 to 433; 445 to 495; 497 to 541). (D) *S. mediterranea* SMED-MT-MMPB and *D. japonica* DJ-MT-MMPB. SMED-MT-MMPB is 636 amino acids in length. The pro-domain region spreads from amino acid 1 to 111. The cystein-switch is detected in position 99 to 105. The presence of a membrane anchor domain (4 to 30) has been predicted. The catalytic domain consists of 159 amino acids (112 to 271) and contains the conserved motif required for the catalytic activity. Downstream this domain (224 to 234) is a flexible proline-rich hinge region (309 to 400) containing unusual repeats (TTTPEP: 316 to 388). Only two hemopexin-like motifs have been detected in the sequence (487 to 534 and 571 to 619).
(TIF)

Figure S3 **Intron-exon organization and phylogeny of the planarian** ***mmp*** **genes.** Exons shown in orange boxes are joined by introns denoted by grey lines. Green boxes represent 3′ UTRs. The number of the exon is indicated in the boxes. The diagram is to scale. Kbp: kilo base pairs. (A) *Smed-mmp1* is organized in 10 exons and 9 introns. (B) *Smed-mmp2* is organized in 10 exons of length similar to those of *Smed-mmp1*, but separated by 9 introns of different length. (C) *Smed-mt-mmpA* shows a genomic organization with 9 exons and 8 introns. (D) *Smed-mt-mmpB* gene is also organized in 9 exons and 8 introns. However, the sizes of the exons, as well as those of the introns, do not coincide with those of *Smed-mt-mmpA.* (E) Amino acid sequences of *S. mediterranea* MMPs were aligned with representative MMP using Muscle. Phylogenetic analysis was performed by RAxML considering a Maximum-likelihood estimation. All three trees were produced on the iTOL web server; the circular main tree shows all the sequences considered on the phylogenetic analysis, all MMP sub-families, where the two small trees are zooming out two specific clusters containing the four *S. mediterranea* MMP proteins. The four tree leaves related to *S. mediterranea* are marked in red on the main tree. Distinct colors were used for the sequences belonging to each of the annotated clusters built upon sets of homologous MMP protein sequences (see color codes on the “MMP sub-families” legend panel on the right). Values shown on the branches of the sub-trees are based on 500 bootstrap replicates. On the main tree bootstrap values below 25% are shown as blue lines and those above 75% are colored in yellow; black lines are for the branches of the tree leaves. Furthermore, HMM profiles for 5 PFAM domains were used to locate them on all the sequences, the corresponding domain structure is drawn on the outer rim of the main tree (underlying black line being proportional to the length of the full protein sequence). *Aedes aegypti* [Aaeg]: MMP19h_Aaeg (gi157110775), MMP24h_Aaeg (gi157110775); *Anolis carolinensis* [Acar]: MMP19hx_Acar (gi327277063), MMP24hx_Acar (gi327271606); *Acromyrmex echinatior* [Aech]: MMP14h_Aech (gi332019904); *Anopheles gambiae* str. PEST [Agam]: MMP2hx_Agam (gi158300836), MMP14x_Agam (gi158286645), MMP17x_Agam (gi158300836), MMP19h_Agam (gi158286645), MMP25x_Agam (gi158298189); *Apis mellifera* [Amel]: MMP19h-14x_Amel (gi328780642); *Ascaris suum* [Asuu]: MMP10h_Asuu (gi324504066), MMP24h_Asuu (gi324507836); *Branchiostoma floridae* [Bflo]: MMP16hx_Bflo (gi260820674); *Bombyx mori* [Bmor]: MMP1hi2_Bmor (gi172356221); *Bos taurus* [Btau]: MMP1p_Btau (gi27806541), MMP1x_Btau (gi359072641), MMP2_Btau (gi27807447), MMP3p_Btau (gi331284133), MMP7p_Btau (gi115496540), MMP8x_Btau (gi359072657), MMP9p_Btau (gi27807437), MMP11x_Btau (gi297485001), MMP12p_Btau (gi331284141), MMP13p_Btau (gi27805999), MMP14p_Btau (gi27806001), MMP15p_Btau (gi300794469), MMP16_Btau (gi300797815), MMP17x_Btau (gi297484686), MMP19p_Btau (gi115496089), MMP20p_Btau (gi27806003), MMP23B_Btau (gi84370137), MMP24x_Btau (gi359071714), MMP25x_Btau (gi297490016), MMP27p_Btau (gi149642799); *Crotalus adamanteus* [Cada]: MMP14h_Cada (gi387017026); *Caiman crocodilus apaporiensis* [Ccro]: MMP20h_Ccro (gi117647501); *Caenorhabditis elegans* [Cele]: MMP13h_Cele (gi3152406), MMP17h_Cele (gi25152490), MMP28x_Cele (gi17558356); *Crassostrea gigas* [Cgig]: MMP17h_Cgig (gi405975212), MMP19Bh_Cgig (gi405975214), MMP19Ah_Cgig (gi405972810), MMP20h_Cgig (gi405978534); *Ciona intestinalis* [Cint]: MMP1h_Cint (gi198420757), MMP16h-14x_Cint (gi198430583), MMP19h-1x_Cint (gi198422839), MMP21hx_Cint (gi198429635), MMP21hx_Cint (gi198429635), MMP24h-1_Cint (gi198420757); *Canis lupus familiaris* [Clup]: MMP1x_Clup (gi345800008), MMP2x_Clup (gi345794260), MMP3p_Clup (gi50950195), MMP7p_Clup (gi337298612), MMP8x_Clup (gi345799783), MMP9p_Clup (gi50978992), MMP11p_Clup (gi337298627), MMP12x_Clup (gi345799781), MMP13x_Clup (gi73955220), MMP14xi2_Clup (gi73962349), MMP15x_Clup (gi345793895), MMP16x_Clup (gi345793184), MMP17x_Clup (gi359322991), MMP19x_Clup (gi345776489), MMP20x_Clup (gi73955232), MMP21x_Clup (gi359323214), MMP23Bx_Clup (gi73956582), MMP24x_Clup (gi73992282), MMP25x_Clup (gi345801954), MMP26x_Clup (gi73988268), MMP27x_Clup (gi73955230), MMP28x_Clup (gi73966944); *Cynops pyrrhogaster* [Cpyr]: MMP21h_Cpyr (gi14787103); *Culex quinquefasciatus* [Cqui]: MMP1h_Cqui (gi170051326), MMP11h_Cqui (gi167881405), MMP19h_Cqui (gi170051326); *Dicentrarchus labrax* [Dlab]: MMP16h_Dlab (gi317419770), MMP21h_Dlab (gi317419524); *Drosophila melanogaster* [Dmel]: MMP1hiJ_Dmel (gi320544352), MMP2h_Dmel (gi45552531), MMP14-1iC_Dmel (gi221468755), MMP15h_Dmel (gi24644242), MMP17-2_Dmel (gi45552531), MMP24h-1iD_Dmel (gi221468756); *Daphnia pulex* [Dpul]: MMP1h_Dpul (gi321455269); *Danio rerio* [Drer]: MMP2_Drer (gi37620187), MMP9p_Drer (gi47085869), MMP10p_Drer (gi94536884), MMP11xB_Drer (gi292625366), MMP11xA_Drer (gi326670156), MMP13x_Drer (gi125838084), MMP14p_Drer (gi35903106), MMP15x_Drer (gi326669661), MMP16x_Drer (gi326664684), MMP17x_Drer (gi326677672), MMP19x_Drer (gi326671556), MMP20x_Drer (gi326674250), MMP21x_Drer (gi326672700), MMP23Bp_Drer (gi62955747), MMP24x_Drer (gi189520793), MMP25Ax_Drer (gi292619299), MMP25Bx_Drer (gi292611844), MMP28x_Drer (gi292621203); *Gallus gallus* [Ggal]: MMP1x_Ggal (gi50731121), MMP2_Ggal (gi45383321), MMP3x_Ggal (gi363729216), MMP7p_Ggal (gi57529313), MMP9p_Ggal (gi45382789), MMP10x_Ggal (gi50731119), MMP11x_Ggal (gi363740125), MMP13x_Ggal (gi363729353), MMP15x_Ggal (gi363737986), MMP16_Ggal (gi45383954), MMP17x_Ggal (gi118098430), MMP23Bx_Ggal (gi50759211), MMP24x_Ggal (gi363741721), MMP27p_Ggal (gi45384534), MMP28x_Ggal (gi363741276); *Harpegnathos saltator* [Hsal]: MMP14h_Hsal (gi307194255); *Homo sapiens* [Hsap]: MMP1p_Hsap (gi4505215), MMP2p_Hsap (gi11342666), MMP3p_Hsap (gi4505217), MMP7p_Hsap (gi4505219), MMP8p_Hsap (gi4505221), MMP9p_Hsap (gi74272287), MMP10p_Hsap (gi4505205), MMP11p_Hsap (gi58331148), MMP12p_Hsap (gi73858572), MMP13p_hsap (gi4505209), MMP14p_Hsap (gi4826834), MMP15p_Hsap (gi4505211), MMP16_Hsap (gi13027802), MMP17_Hsap (gi112382270), MMP19_Hsap (gi4505213), MMP20p_Hsap (gi45359865), MMP21p_Hsap (gi22218341), MMP23Bp_Hsap (gi5902004), MMP24p_Hsap (gi5729929), MMP25p_Hsap (gi11968059), MMP26_Hsap (gi119589223), MMP27p_Hsap (gi73808268), MMP28pi1_Hsap (gi13236530); *Ictalurus punctatus* [Ipun]: MMP13h_Ipun (gi291195937), MMP19h-13p_Ipun (gi319072680); *Ixodes scapularis* [Isca]: MMP19hx_Isca (gi242000886); *Macaca mulatta* [Mmul]: MMP1xi2_Mmul (gi109108464), MMP2x_Mmul (gi109128539), MMP3xi1_Mmul (gi109108468), MMP7x_Mmul (gi297269060), MMP8x_Mmul (gi297269064), MMP9x_Mmul (gi109091737), MMP10x_Mmul (gi109109582), MMP11x_Mmul (gi109094826), MMP12x_Mmul (gi109108472), MMP13xi4_Mmul (gi109108476), MMP14x_Mmul (gi109082872), MMP15x_Mmul (gi109128713), MMP16xi2_Mmul (gi109086884), MMP17x_Mmul (gi297263898), MMP19x_Mmul (gi109097139), MMP20x_Mmul (gi109108456), MMP21x_Mmul (gi109090903), MMP23Bx_Mmul (gi108995571), MMP24x_Mmul (gi297259948), MMP25x_Mmul (gi109127347), MMP26x_Mmul (gi297268618), MMP27x_Mmul (gi109108458), MMP28xi1_Mmul (gi297272410); *Mus musculus* [Mmus]: MMP1p_Mmus (gi14030785), MMP2_Mmus (gi6678902), MMP3p_Mmus (gi6754714), MMP7p_Mmus (gi111955033), MMP8p_Mmus (gi160333381), MMP9p_Mmus (gi7305277), MMP10p_Mmus (gi9506899), MMP11p_Mmus (gi6678894), MMP12p_Mmus (gi115392138), MMP13p_Mmus (gi6678896), MMP14p_Mmus (gi188528637), MMP15p_Mmus (gi6678900), MMP16p_Mmus (gi40254544), MMP17p_Mmus (gi31543257), MMP19p_Mmus (gi10946772), MMP20p_Mmus (gi7305275), MMP21p_Mmus (gi23308683), MMP23B_Mmus (gi6754710), MMP24p_Mmus (gi115495469), MMP25p_Mmus (gi75677470), MMP27_Mmus (gi71892410), MMP28pi2_Mmus (gi30348964); *Nasonia vitripennis* [Nvir]: MMP9h_Nvir (gi60207620); *Notophthalmus viridescens* [Nvit]: MMP1h_Nvit (gi301176641); *Oryctolagus cuniculus* [Ocun]: MMP19hx_Ocun (gi291389397); *Oryzias latipes* [Olat]: MMP19h_Olat (gi157278171); *Oncorhynchus mykiss* [Omyk]: MMP19h_Omyk (gi185134636); *Pan troglodytes* [Ptro]: MMP1xi3_Ptro (gi114640096), MMP2x_Ptro (gi332845945), MMP3xi4_Ptro (gi114640107), MMP7xi2_Ptro (gi114640081), MMP8x_Ptro (gi332837587), MMP9xi1_Ptro (gi332858607), MMP10xi4_Ptro (gi114640098), MMP11x_Ptro (gi114685393), MMP12x_Ptro (gi114640115), MMP13xi2_Ptro (gi114640119), MMP14xi4_Ptro (gi114652041), MMP15xi1_Ptro (gi332846033), MMP16xi2_Ptro (gi114620829), MMP17x_Ptro (gi114647860), MMP19x_Ptro (gi332839031), MMP20x_Ptro (gi114640083), MMP21x_Ptro (gi332835329), MMP23Bx_Ptro (gi332807452), MMP24x_Ptro (gi332858203), MMP25x_Ptro (gi332845157), MMP26x_Ptro (gi114635797), MMP27x_Ptro (gi55637151), MMP28x_Ptro (gi332848066); *Rhinolophus ferrumequinum* [Rfer]: MMP24hxp_Rfer (gi184185552); *Rattus norvegicus* [Rnor]: MMP1p_Rnor (gi197384336), MMP2_Rnor (gi146262019), MMP3p_Rnor (gi19424170), MMP7p_Rnor (gi6981214), MMP8p_Rnor (gi11560008), MMP9p_Rnor (gi13591993), MMP10p_Rnor (gi19424154), MMP11_Rnor (gi6981212), MMP12p_Rnor (gi16758852), MMP13p_Rnor (gi213972541), MMP14p_Rnor (gi13591995), MMP15p_Rnor (gi157818219), MMP16p_Rnor (gi18158443), MMP17p_Rnor (gi157786912), MMP19p_Rnor (gi157820645), MMP20p_Rnor (gi157817855), MMP21p_Rnor (gi157821123), MMP23B_Rnor (gi16758396), MMP25_Rnor (gi293351330), MMP27p_Rnor (gi157818495), MMP28p_Rnor (gi120586977); *Rhabdophis tigrinus tigrinus* [Rtig]: MMP9h_Rtig (gi171702397); *Sorex araneus* [Sara]: MMP24hxp_Sara (gi190402260); *Serinus canaria* [Scan]: MMP2h_Scan (gi148727872); *Sarcophilus harrisii* [Shar]: MMP21hx_Shar (gi395501358); *Saccoglossus kowalevskii* [Skow]: MMP19hpi1_Skow (gi291237328), MMP24hxp_Skow (gi291237326); *Schistosoma mansoni* [Sman]: MMP15h_Sman (gi256081444); *Strongylocentrotus purpuratus* [Spur]: MMP14p_Spur (gi75832177), MMP16h_Spur (gi62005762), MMP19h-16p_Spur (gi75832168); *Salmo salar* [Ssal]: MMP19h_Ssal (gi213514500); *Sus scrofa* [Sscr]: MMP19hx_Sscr (gi335288059); *Tribolium castaneum* [Tcas]: MMP1hi2_Tcas (gi255958234), MMP2h_Tcas (gi270012816); *Taeniopygia guttata* [Tgut]: MMP19h-27x_Tgut (gi224043580); *Xenopus laevis* [Xlae]: MMP3hp_Xlae (gi75863761), MMP7h_Xlae (gi46560178), MMP8hp_Xlae (gi148227598), MMP11h_Xlae (gi414918), MMP13hp_Xlae (gi155369235), MMP20h_Xlae (gi117647503), MMP21hp_Xlae (gi147904008), MMP24hp_Xlae (gi148232353), MMP28hp_Xlae (gi288557361); *Xenopus* (*Silurana*) *tropicalis* [Xtro]: MMP15h_Xtro (gi62860164), MMP19hx_Xtro (gi301620474), MMP23Bh_Xtro (gi301611320); and *Schmidtea mediterranea* [Smed] MMPs described on this paper. Letters after the MMP sub-family number on the sequence names have the following meaning: ‘h’, for homologous to UniGene sequences; ‘x’ for predicted/hypothetical protein sequences (XP RefSeq entries); ‘p’ for precursor/preprotein (only for NP RefSeq sequences); ‘i’ for annotated/putative isoform.
(TIF)

Figure S4 **Expression pattern of** ***mmp1*** **and** ***mmp2*** **in** ***S. mediterranea*** **.** WISH depicting the distribution of *Smed-mmp1* and *Smed-mmp2* expression on intact planarians. (A) *Smed-mmp1* antisense probe. (B) *Smed-mmp2* antisense probe. Anterior to the left. To characterize further the cell types expressing *Smed-mmp1* and *Smed-mmp2*, ISH experiments were carried out on wax longitudinal sections. (C) Some *Smed-mmp1*- and *Smed-mmp2*-positive cells, localized in subepidermal position, are detected. These cells appear rather large, slightly polygonal or bottle-shaped, with a round nucleus and a well-defined nucleolus. ep: epithelium. (D) After ISH, Heidenhain’s azan stain (0,5% w/v aniline blue, 2 g w/v orange G, 8% v/v acetic acid) identifies *Smed-mmp1*-expressing cells as a subpopulation of cyanophilic secretory cells. Dorsal to the top. (E) *Smed-mmp1* whole mount in situ hybridization of planarian fragments, analyzed at 1 and 5 days of regeneration, respectively. The ring-shaped expression pattern does not change significantly in trunk fragments, while some labelled cells begin to be detected in head and tail fragments only after 4–5 days of regeneration. Anterior is up. Scale bars: 1 mm in A and E; 50 µm in C; 200 µm in D.
(TIF)

Figure S5 **Expression of** ***mt-mmpA*** **in** ***S. mediterranea.*** (A) WISH with antisense *Smed-mt-mmpA* probe in intact planarians detects widespread expression, while lack of signal with sense *Smed-mt-mmpA* probe confirms the specificity of the expression. Anterior is up. Scale bar: 1 mm. (B) Real Time RT-PCR analysis of *Smed-mt-mmpA* expression level in lethally-irradiated planarians, sacrificed at different days after exposure. *Smed-mcm2* is analyzed as a control. The expression level is indicated in relative units, assuming the value of non-irradiated planarians as unitary. Each value is the mean ± s.d. of three independent samples, analyzed in duplicate. Consistent with the data obtained in *D. japonica*, the level of *Smed-mt-mmpA* transcripts does not change significantly after irradiation, while the expression level of the stem cell marker *Smed-mcm2* appears dramatically downregulated, demonstrating the effectiveness of irradiation. c: non-irradiated planarians. Significant differences in the expression level of *Smed-mcm2* between irradiated and non-irradiated planarians were detected using the analysis of variance (ANOVA) ***P = 0.0001.
(TIF)

Figure S6 ***Smed-mmp1(RNAi)*** **does not inhibit regeneration.** Bright-field images of head and tail fragments of water-injected controls and following *Smed-mmp1(RNAi)*, as visualized at 3 days after amputation in *S. mediterranea*. Neither blastema formation nor blastema size are altered in *Smed-mmp1(RNAi)* regenerating head and tail fragments. Anterior is up. Scale bars: 1 mm.
(TIF)

Figure S7 **TUNEL assay after** ***Dj-mmp1(RNAi)*** **in** ***D. japonica*** **.** Apoptotic cells are detected as red spots in longitudinal cryosections, visualized at the pre-pharyngeal level in an intact *D. japonica*. (A) A water-injected control. (B) A *Dj-mmp1(RNAi)* planarian. e: eye. Anterior is to the left. Consistent with the data obtained in *S. mediterranea,* a decrease in the number of apoptotic cells can be observed in *D. japonica* after *Dj-mmp1(RNAi)*. Scale bar: 1 mm.
(TIF)

Figure S8 ***Smed-mt-mmpB(RNAi)*** **does not produce any phenotype.** (**A**) Bright-field images of head and tail fragments at 5 days of regeneration in *S. mediterranea*. Control: *β-gal(RNAi)*, *mt-mmpA/mt-mmpB(RNAi)*: simultaneous *Smed-mt-mmpA(RNAi)* and *Smed-mt-mmpB(RNAi)*. No morphological difference between the *β-gal(RNAi)* controls and *Smed-mt-mmpB(RNAi)* animals is detected. Simultaneous *Smed-mt-mmpA(RNAi)* and *Smed-mt-mmpB(RNAi)* only produces *mt-mmpA(RNAi)* phenotypes. (B) Expression level of different markers (*Smed-mcm2, Smed-prog1*, *Smed-AGAT1*, *Smed-syt*) after *Smed-mt-mmpB(RNAi)* and simultaneous *Smed-mt-mmpA(RNAi)* and *Smed-mt-mmpB(RNAi)*, analyzed by Real Time RT-PCR. No significant variation in the expression level of different markers is observed. In the Real Time RT-PCR experiments the expression level is indicated in relative units, assuming as unitary the value of the controls. Each value is the mean ± s.d. of three independent samples, carried out in duplicate. c: *β-gal(RNAi)*; *mt-mmpA/mt-mmpB(RNAi)*: *Smed-mt-mmpA(RNAi) and Smed-mt-mmpB(RNAi)*; *mt-mmpB(RNAi): Smed-mt-mmpB(RNAi).* Scale bars: 1 mm.
(TIF)

Figure S9 **Characterization of** ***Smed-mt-mmpA(RNAi)*** **phenotypes in intact** ***S. mediterranea*** **.** (A) Bright-field image of a *β-gal(RNAi)* control and a *Dj-mt-mmpA(RNAi)* planarian, at 8 days after the first injection. Dorsal view, anterior is up. Scale bar: 1 mm. (B) Real Time RT-PCR analysis reveals that the expression level of both *Smedwi-1* and *Smed-prog-1* is not affected by *Smed-mt-mmpA(RNAi)*. (C) A significant decrease in the expression level of differentiated tissue markers (*Smed-AGAT1* and *Smed-syt*) has been observed between *Smed-mt-mmpA(RNAi)* planarians and controls by Real Time RT-PCR. (D) Activation of *Smed-dap1* expression is observed in *Smed-mt-mmpA(RNAi)* animals compared to controls. In the Real Time RT-PCR experiments the expression level is indicated in relative units, assuming as unitary the value of the controls. Each value is the mean ± s.d. of three independent samples, carried out in duplicate. Samples were compared using the unpaired t-test. **P<0.001. c: *β-gal(RNAi)* control; *mt-mmpA(RNAi)*: *Smed-mt-mmpA(RNAi)*.
(TIF)

Figure S10 **Characterization of** ***mt-mmpA(RNAi)*** **phenotype in** ***D. japonica,*** **at 5 days of regeneration.** (A) Bright–field images of head and tail fragments, after two rounds of dsRNA injections and amputation. Control: *β-gal(RNAi)* fragments. Following *Dj-mt-mmpA*(*RNAi)* the fragments show a significantly reduced blastema. Relative blastema size between *Dj-mt-mmpA(RNAi)* and *β-gal(RNAi)* fragments was calculated as percentage of the whole fragment, considered as unitary. (B) Expression level of different markers (*Dj-mcm2, Dj-inx11*, *Dj-bruli, Dj-inx1, Dj-ahnack, Dj-syt, Dj-gad* and *Dj-dap1*) after *Dj-mt-mmpA(RNAi)*, analyzed by Real Time RT-PCR. No significant variation in the expression level of different markers was observed. In the Real Time RT-PCR experiments the expression level is indicated in relative units, assuming as unitary the value of the controls. Each value is the mean ± s.d. of three independent samples, carried out in duplicate. c: *β-gal(RNAi)*; *mt-mmpA(RNAi)*: *Dj-mt-mmpA(RNAi)*.
(TIF)

Figure S11 **Immunostaining with anti-H3P antibody in** ***Smed-mt-mmpA(RNAi)*** **fragments, at 6 days of head regeneration.** (A) *β-gal(RNAi)* control. (B) *Smed-mt-mmpA(RNAi)*. After S*med-mt-mmpA(RNAi)* silencing the regenerative blastema results reduced in size and a smaller number of anti-H3P-positive cells migrated into the blastema with respect to the control. Scale bar: 200 µm.
(TIF)
