# Supplementary figures and images for: Planarians as a Model to Assess In Vivo the Role of Matrix Metalloproteinase Genes during Homeostasis and Regeneration
Source: PLoS One. 2013 Feb 6;8(2):e55649. doi: 10.1371/journal.pone.0055649 (PMC3566077; doi:10.1371/journal.pone.0055649)

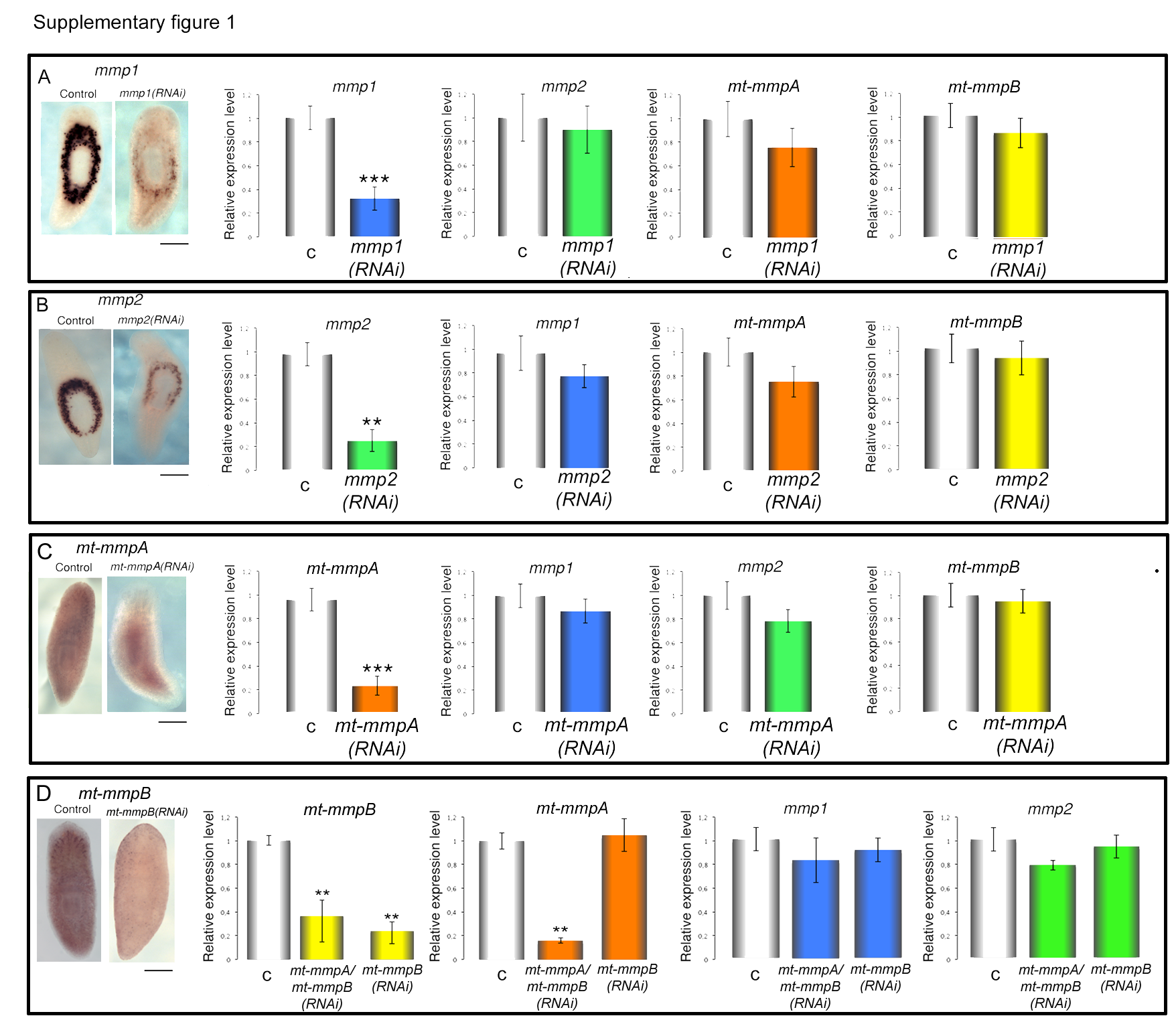

Supplement: Figure S1 — Analysis of efficiency and specificity of each RNAi silencing in S. mediterranea . (A) Smed-mmp1 expression level as detected by WISH and Real Time RT-PCR in controls and Smed-mmp1(RNAi) demonstrates the efficiency of the treatment. Moreover, Smed-mmp1(RNAi) does not affect the expression level of Smed-mmp2, Smed-mt-mmpA and Smed-mt-mmpB. (B) Smed-mmp2 expression level as detected by WISH and Real Time RT-PCR in controls and Smed-mmp2(RNAi) animals. Moreover, Smed-mmp2(RNAi) does not affect the expression level of Smed-mmp1, Smed-mt-mmpA and Smed-mt-mmpB. (C) Smed-mt-mmpA expression level as detected by WISH and Real Time RT-PCR in controls and Smed-mt-mmpA(RNAi) animals. Moreover, Smed-mt-mmpA(RNAi) does not affect the expression level of Smed-mmp1, Smed-mmp2 and Smed-mt-mmpB. (D) Smed-mt-mmpB expression level as detected by WISH and Real Time RT-PCR in controls and Smed-mt-mmpB(RNAi) animals. Moreover, Smed-mt-mmpB(RNAi) does not affect the expression level of Smed-mmp1, Smed-mmp2 and Smed-mt-mmpA. The coinjection of Smed-mt-mmpA and Smed-mt-mmpB does not affect the expression level both of Smed-mmp1 and Smed-mmp2. In the Real Time RT-PCR experiments the expression level is indicated in relative units, assuming as unitary the value of the controls. Each value is the mean ± s.d. of three independent samples, carried out in duplicate. (unpaired t-test) **P<0.001, ***P<0.0001. c: β-gal(RNAi) controls; mmp1(RNAi): Smed-mmp1(RNAi); mmp2(RNAi): Smed-mmp2(RNAi); mt-mmpA(RNAi): Smed-mt-mmpA(RNAi); mt-mmpB(RNAi): Smed-mt-mmpB(RNAi); mt-mmpA/mt-mmpB(RNAi): Smed-mt-mmpA and Smed-mt-mmpB(RNAi). Scale bars: 1 mm. (TIF) [file pone.0055649.s001.tif]

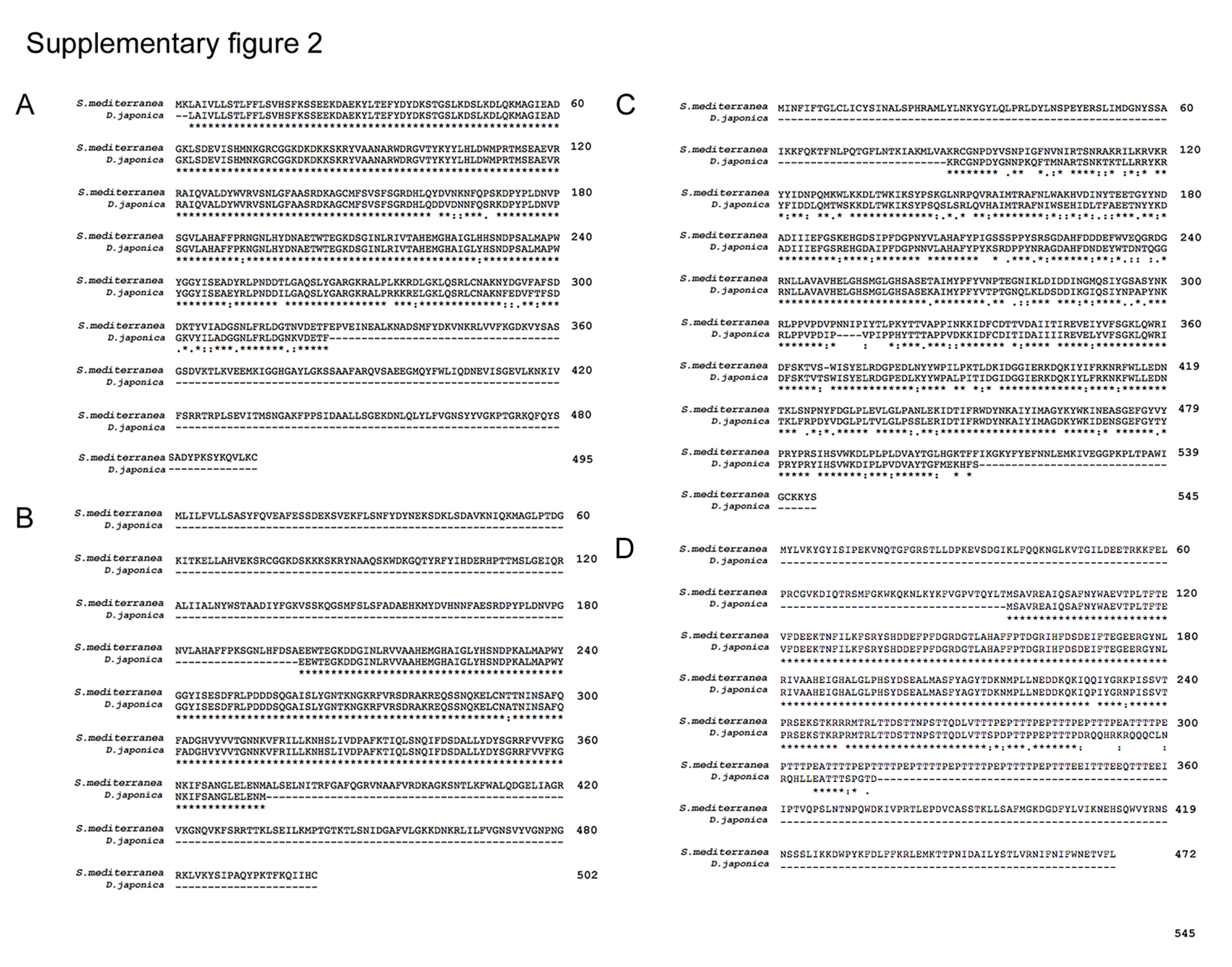

Supplement: Figure S2 — Amino acid sequence alignment of MMP1, MMP2, MT-MMPA and MT-MMPB from S. mediterranea and D. japonica . (A) S. mediterranea SMED-MMP1 and D. japonica DJ-MMP1. SMED-MMP1 is 495 amino acids in length. The pro-domain consists of 80 amino acids (1 to 80) and contains the pro-peptide cleavage site in position 18 and an atypical cysteine switch (GRCGGKD, 73 to 80). The catalytic domain consists of 175 amino acids (91 to 266) and contains the conserved HEXXHXXGXXH motif (221 to 231). The typical methionine turn is in position 237. (B) S. mediterranea SMED-MMP2 and D. japonica DJ-MMP2. SMED-MMP2 is 502 amino acids in length. The pro-domain (1 to 79) contains an atypical cysteine switch (SRCGGKD, 72 to 79). The propeptide cleavage site is in position 17. The catalytic domain (90 to 265) includes the HEXXHXXGXXH motif (218 to 228). Methionine turn is in position 236. (C) S. mediterranea SMED-MT-MMPA and D. japonica DJ-MT-MMPA. SMED-MT-MMPA is 545 amino acids in length. The pro-domain region spreads from amino acid 1 to 93. The cystein-switch is detected in position 87 to 93. The presence of a membrane anchor domain (1 to 21) and a furin cleavage site (ILKRVKR: 114 to 120) has been predicted. The catalytic domain consists of 169 amino acids (126 to 295) and contains the conserved motif required for the catalytic activity. Downstream this domain (249 to 259) is a flexible proline-rich hinge region (303 to 326) followed by four hemopexin-like motifs (338 to 389; 394 to 433; 445 to 495; 497 to 541). (D) S. mediterranea SMED-MT-MMPB and D. japonica DJ-MT-MMPB. SMED-MT-MMPB is 636 amino acids in length. The pro-domain region spreads from amino acid 1 to 111. The cystein-switch is detected in position 99 to 105. The presence of a membrane anchor domain (4 to 30) has been predicted. The catalytic domain consists of 159 amino acids (112 to 271) and contains the conserved motif required for the catalytic activity. Downstream this domain (224 to 234) is a flexible proline-rich hinge reg [file pone.0055649.s002.tif]

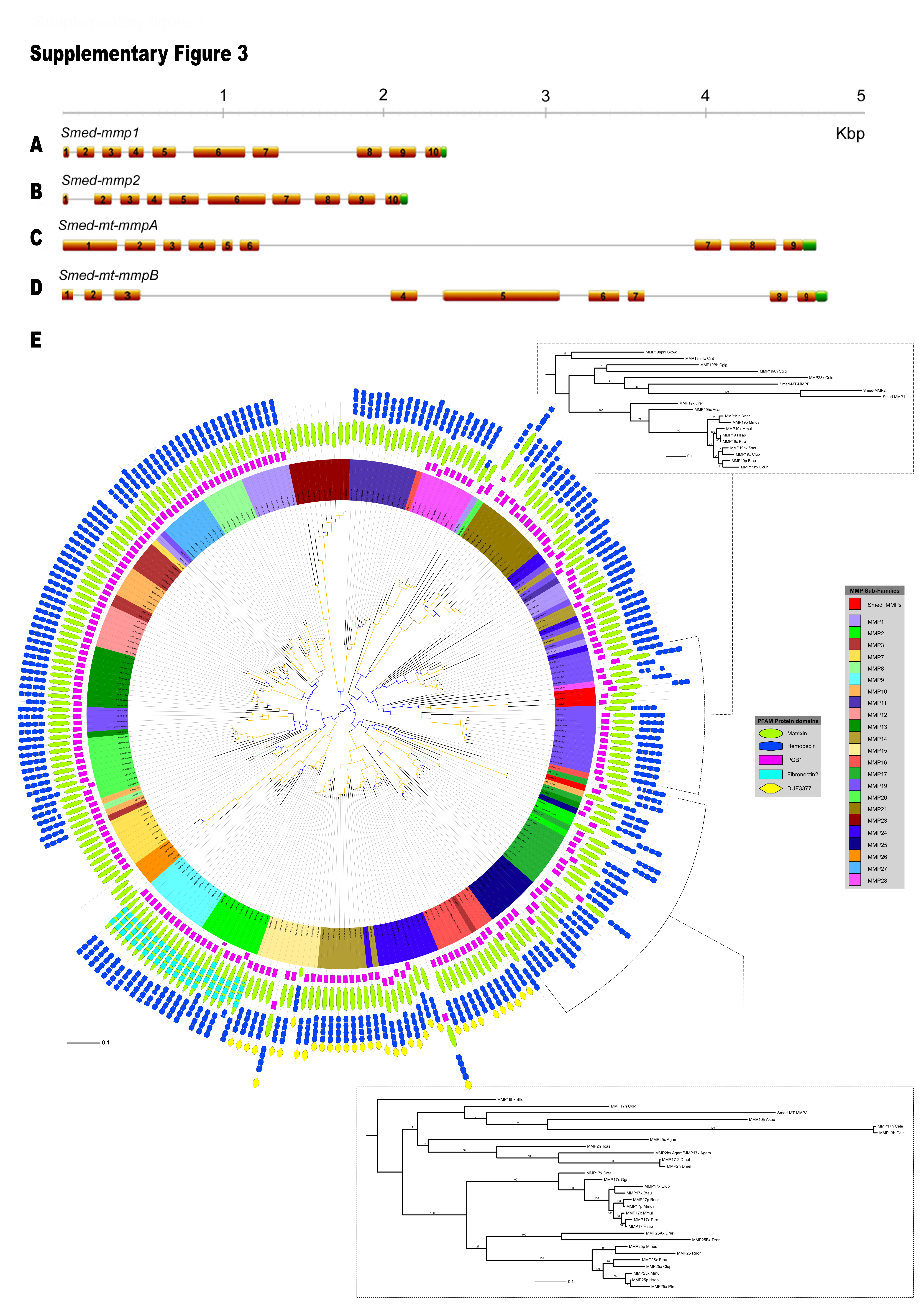

Supplement: Figure S3 — Intron-exon organization and phylogeny of the planarian mmp genes. Exons shown in orange boxes are joined by introns denoted by grey lines. Green boxes represent 3′ UTRs. The number of the exon is indicated in the boxes. The diagram is to scale. Kbp: kilo base pairs. (A) Smed-mmp1 is organized in 10 exons and 9 introns. (B) Smed-mmp2 is organized in 10 exons of length similar to those of Smed-mmp1, but separated by 9 introns of different length. (C) Smed-mt-mmpA shows a genomic organization with 9 exons and 8 introns. (D) Smed-mt-mmpB gene is also organized in 9 exons and 8 introns. However, the sizes of the exons, as well as those of the introns, do not coincide with those of Smed-mt-mmpA. (E) Amino acid sequences of S. mediterranea MMPs were aligned with representative MMP using Muscle. Phylogenetic analysis was performed by RAxML considering a Maximum-likelihood estimation. All three trees were produced on the iTOL web server; the circular main tree shows all the sequences considered on the phylogenetic analysis, all MMP sub-families, where the two small trees are zooming out two specific clusters containing the four S. mediterranea MMP proteins. The four tree leaves related to S. mediterranea are marked in red on the main tree. Distinct colors were used for the sequences belonging to each of the annotated clusters built upon sets of homologous MMP protein sequences (see color codes on the “MMP sub-families” legend panel on the right). Values shown on the branches of the sub-trees are based on 500 bootstrap replicates. On the main tree bootstrap values below 25% are shown as blue lines and those above 75% are colored in yellow; black lines are for the branches of the tree leaves. Furthermore, HMM profiles for 5 PFAM domains were used to locate them on all the sequences, the corresponding domain structure is drawn on the outer rim of the main tree (underlying black line being proportional to the length of the full protein sequence). Aedes aegypti [Aaeg]: MMP19h_Aa [file pone.0055649.s003.tif]

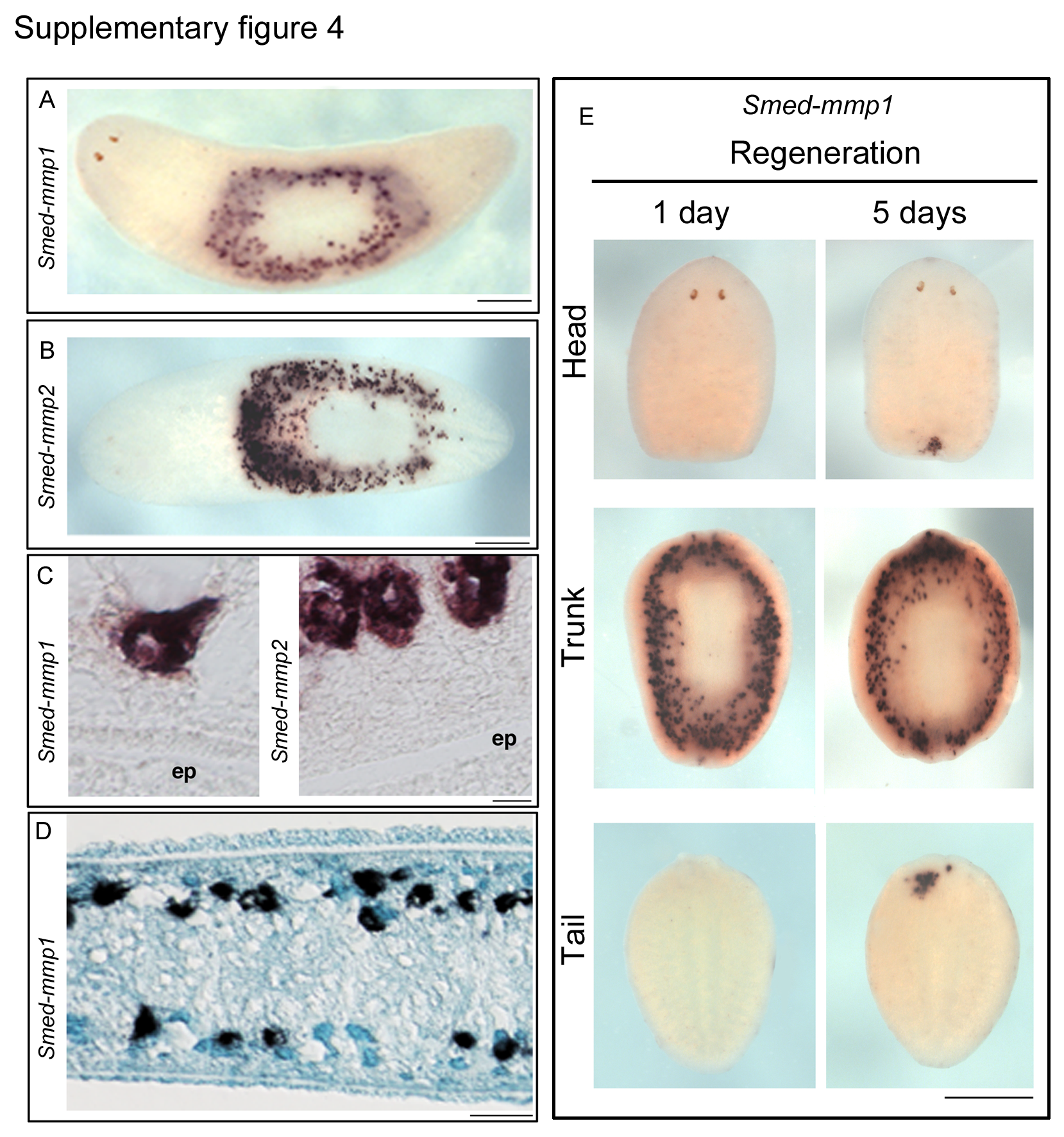

Supplement: Figure S4 — Expression pattern of mmp1 and mmp2 in S. mediterranea . WISH depicting the distribution of Smed-mmp1 and Smed-mmp2 expression on intact planarians. (A) Smed-mmp1 antisense probe. (B) Smed-mmp2 antisense probe. Anterior to the left. To characterize further the cell types expressing Smed-mmp1 and Smed-mmp2, ISH experiments were carried out on wax longitudinal sections. (C) Some Smed-mmp1- and Smed-mmp2-positive cells, localized in subepidermal position, are detected. These cells appear rather large, slightly polygonal or bottle-shaped, with a round nucleus and a well-defined nucleolus. ep: epithelium. (D) After ISH, Heidenhain’s azan stain (0,5% w/v aniline blue, 2 g w/v orange G, 8% v/v acetic acid) identifies Smed-mmp1-expressing cells as a subpopulation of cyanophilic secretory cells. Dorsal to the top. (E) Smed-mmp1 whole mount in situ hybridization of planarian fragments, analyzed at 1 and 5 days of regeneration, respectively. The ring-shaped expression pattern does not change significantly in trunk fragments, while some labelled cells begin to be detected in head and tail fragments only after 4–5 days of regeneration. Anterior is up. Scale bars: 1 mm in A and E; 50 µm in C; 200 µm in D. (TIF) [file pone.0055649.s004.tif]

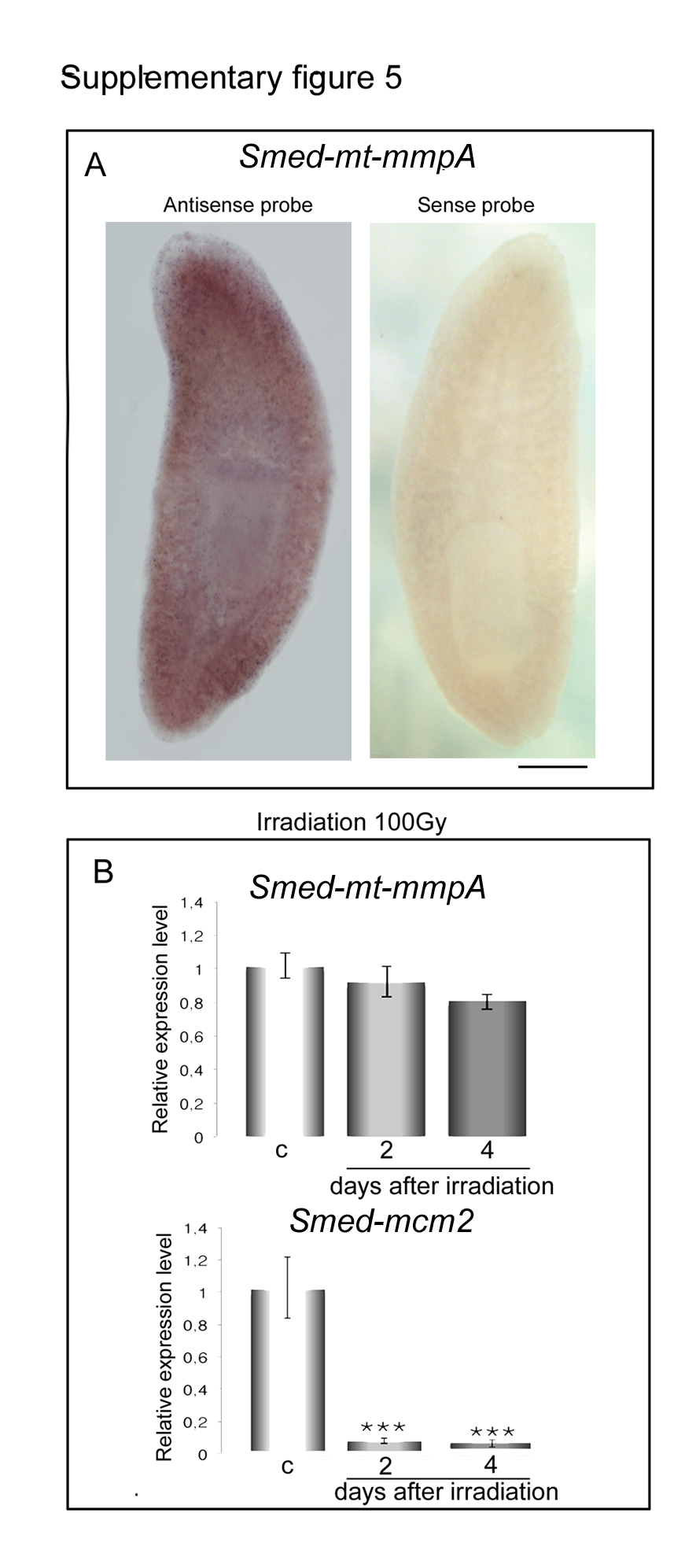

Supplement: Figure S5 — Expression of mt-mmpA in S. mediterranea. (A) WISH with antisense Smed-mt-mmpA probe in intact planarians detects widespread expression, while lack of signal with sense Smed-mt-mmpA probe confirms the specificity of the expression. Anterior is up. Scale bar: 1 mm. (B) Real Time RT-PCR analysis of Smed-mt-mmpA expression level in lethally-irradiated planarians, sacrificed at different days after exposure. Smed-mcm2 is analyzed as a control. The expression level is indicated in relative units, assuming the value of non-irradiated planarians as unitary. Each value is the mean ± s.d. of three independent samples, analyzed in duplicate. Consistent with the data obtained in D. japonica, the level of Smed-mt-mmpA transcripts does not change significantly after irradiation, while the expression level of the stem cell marker Smed-mcm2 appears dramatically downregulated, demonstrating the effectiveness of irradiation. c: non-irradiated planarians. Significant differences in the expression level of Smed-mcm2 between irradiated and non-irradiated planarians were detected using the analysis of variance (ANOVA) ***P = 0.0001. (TIF) [file pone.0055649.s005.tif]

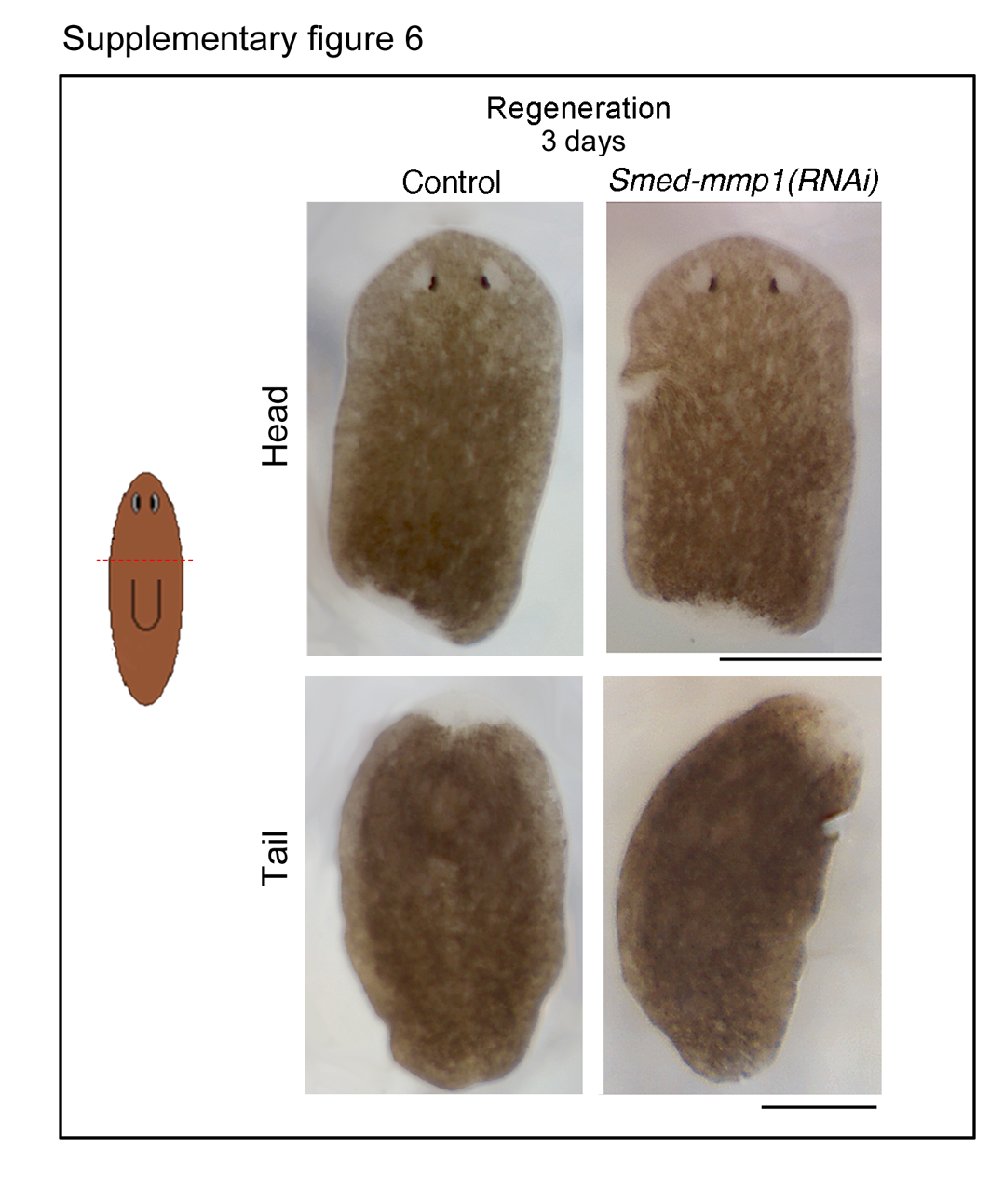

Supplement: Figure S6 — Smed-mmp1(RNAi) does not inhibit regeneration. Bright-field images of head and tail fragments of water-injected controls and following Smed-mmp1(RNAi), as visualized at 3 days after amputation in S. mediterranea. Neither blastema formation nor blastema size are altered in Smed-mmp1(RNAi) regenerating head and tail fragments. Anterior is up. Scale bars: 1 mm. (TIF) [file pone.0055649.s006.tif]

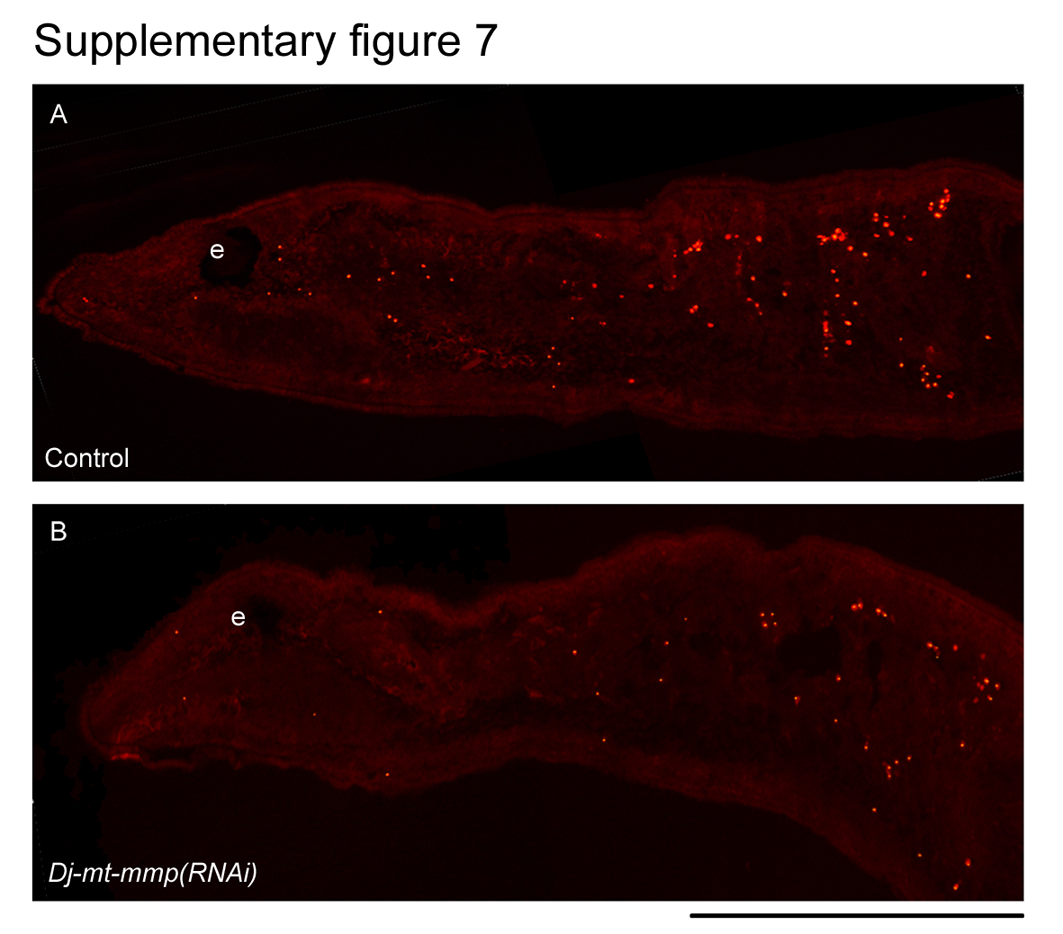

Supplement: Figure S7 — TUNEL assay after Dj-mmp1(RNAi) in D. japonica . Apoptotic cells are detected as red spots in longitudinal cryosections, visualized at the pre-pharyngeal level in an intact D. japonica. (A) A water-injected control. (B) A Dj-mmp1(RNAi) planarian. e: eye. Anterior is to the left. Consistent with the data obtained in S. mediterranea, a decrease in the number of apoptotic cells can be observed in D. japonica after Dj-mmp1(RNAi). Scale bar: 1 mm. (TIF) [file pone.0055649.s007.tif]

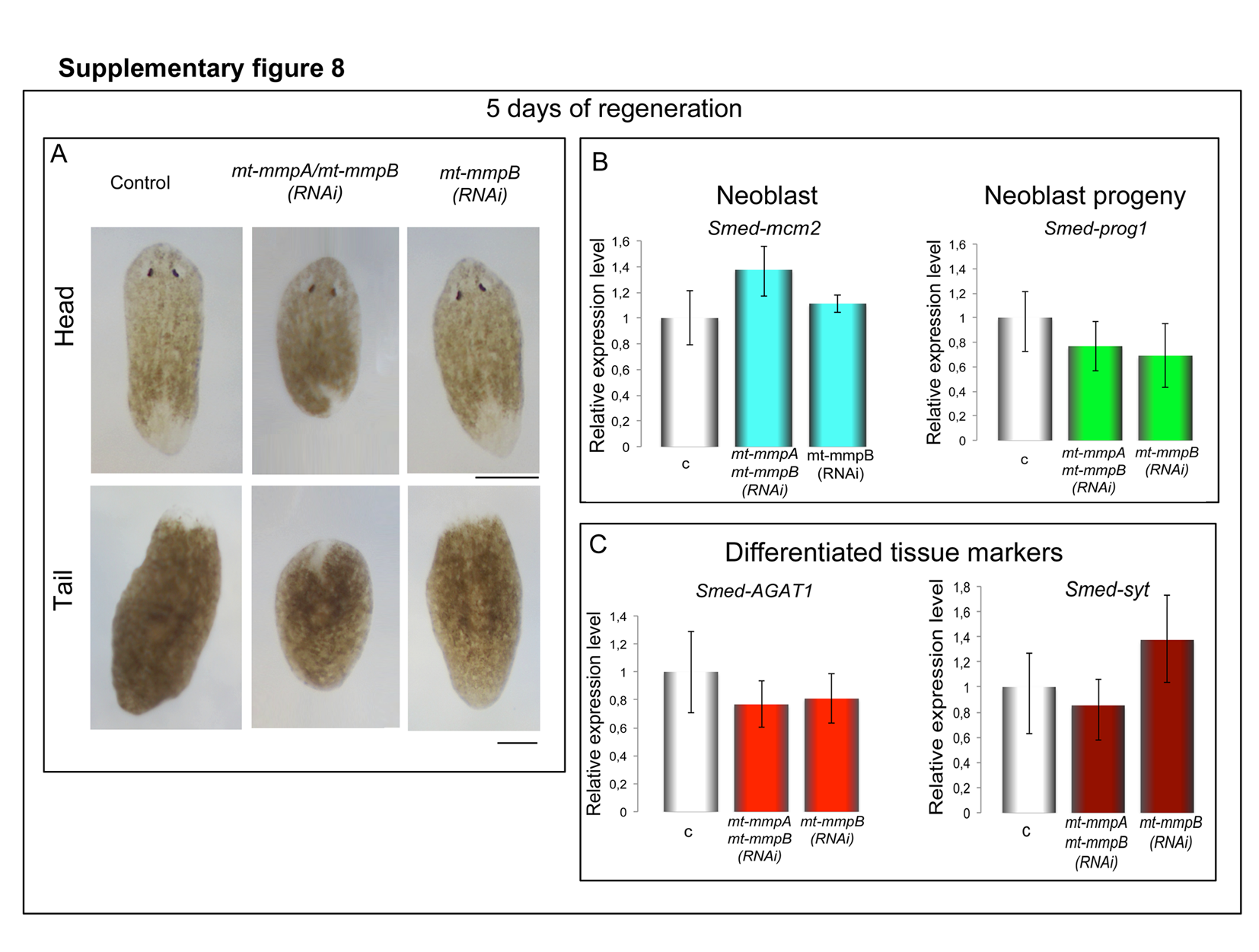

Supplement: Figure S8 — Smed-mt-mmpB(RNAi) does not produce any phenotype. (A) Bright-field images of head and tail fragments at 5 days of regeneration in S. mediterranea. Control: β-gal(RNAi), mt-mmpA/mt-mmpB(RNAi): simultaneous Smed-mt-mmpA(RNAi) and Smed-mt-mmpB(RNAi). No morphological difference between the β-gal(RNAi) controls and Smed-mt-mmpB(RNAi) animals is detected. Simultaneous Smed-mt-mmpA(RNAi) and Smed-mt-mmpB(RNAi) only produces mt-mmpA(RNAi) phenotypes. (B) Expression level of different markers (Smed-mcm2, Smed-prog1, Smed-AGAT1, Smed-syt) after Smed-mt-mmpB(RNAi) and simultaneous Smed-mt-mmpA(RNAi) and Smed-mt-mmpB(RNAi), analyzed by Real Time RT-PCR. No significant variation in the expression level of different markers is observed. In the Real Time RT-PCR experiments the expression level is indicated in relative units, assuming as unitary the value of the controls. Each value is the mean ± s.d. of three independent samples, carried out in duplicate. c: β-gal(RNAi); mt-mmpA/mt-mmpB(RNAi): Smed-mt-mmpA(RNAi) and Smed-mt-mmpB(RNAi); mt-mmpB(RNAi): Smed-mt-mmpB(RNAi). Scale bars: 1 mm. (TIF) [file pone.0055649.s008.tif]

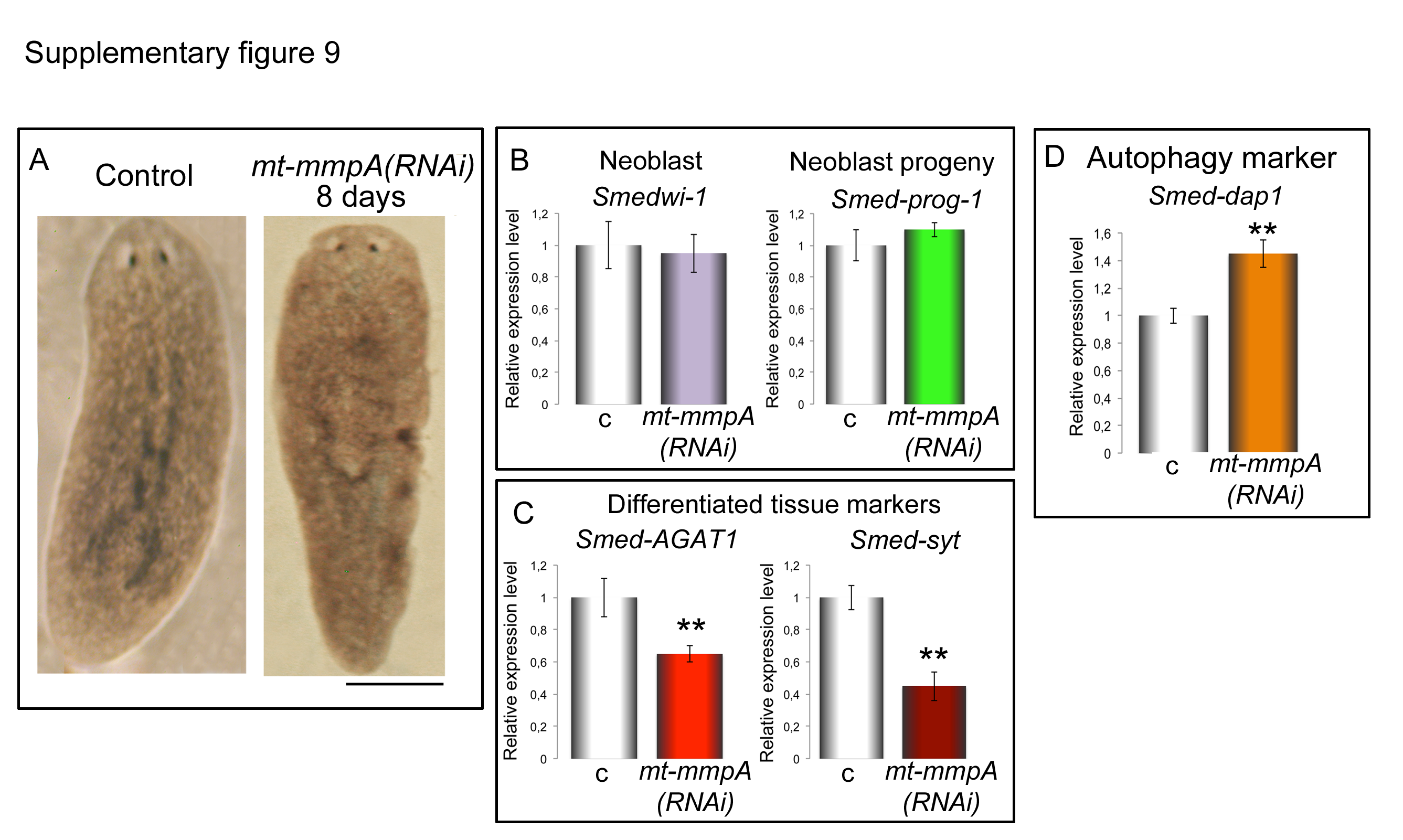

Supplement: Figure S9 — Characterization of Smed-mt-mmpA(RNAi) phenotypes in intact S. mediterranea . (A) Bright-field image of a β-gal(RNAi) control and a Dj-mt-mmpA(RNAi) planarian, at 8 days after the first injection. Dorsal view, anterior is up. Scale bar: 1 mm. (B) Real Time RT-PCR analysis reveals that the expression level of both Smedwi-1 and Smed-prog-1 is not affected by Smed-mt-mmpA(RNAi). (C) A significant decrease in the expression level of differentiated tissue markers (Smed-AGAT1 and Smed-syt) has been observed between Smed-mt-mmpA(RNAi) planarians and controls by Real Time RT-PCR. (D) Activation of Smed-dap1 expression is observed in Smed-mt-mmpA(RNAi) animals compared to controls. In the Real Time RT-PCR experiments the expression level is indicated in relative units, assuming as unitary the value of the controls. Each value is the mean ± s.d. of three independent samples, carried out in duplicate. Samples were compared using the unpaired t-test. **P<0.001. c: β-gal(RNAi) control; mt-mmpA(RNAi): Smed-mt-mmpA(RNAi). (TIF) [file pone.0055649.s009.tif]

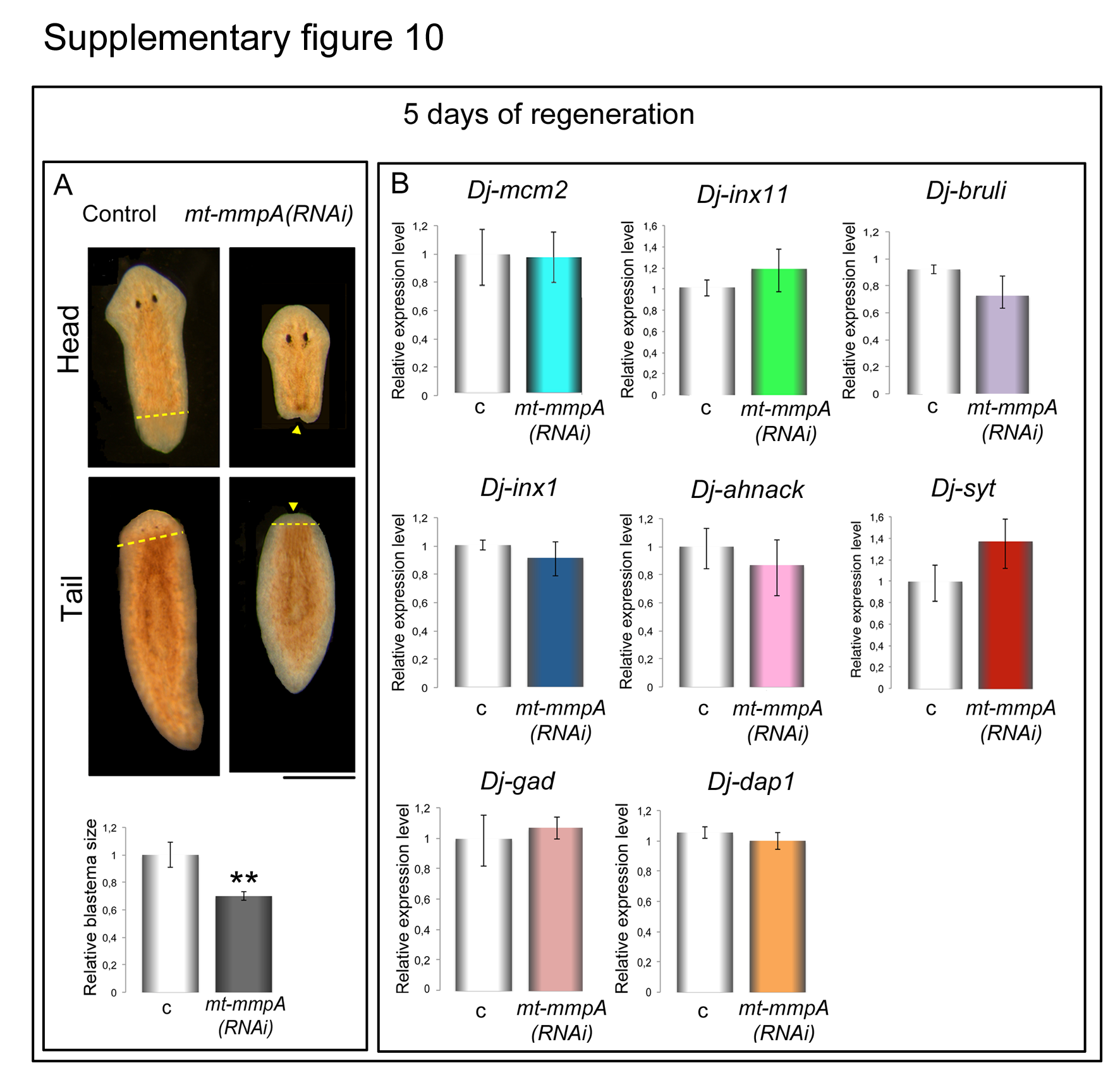

Supplement: Figure S10 — Characterization of mt-mmpA(RNAi) phenotype in D. japonica, at 5 days of regeneration. (A) Bright–field images of head and tail fragments, after two rounds of dsRNA injections and amputation. Control: β-gal(RNAi) fragments. Following Dj-mt-mmpA(RNAi) the fragments show a significantly reduced blastema. Relative blastema size between Dj-mt-mmpA(RNAi) and β-gal(RNAi) fragments was calculated as percentage of the whole fragment, considered as unitary. (B) Expression level of different markers (Dj-mcm2, Dj-inx11, Dj-bruli, Dj-inx1, Dj-ahnack, Dj-syt, Dj-gad and Dj-dap1) after Dj-mt-mmpA(RNAi), analyzed by Real Time RT-PCR. No significant variation in the expression level of different markers was observed. In the Real Time RT-PCR experiments the expression level is indicated in relative units, assuming as unitary the value of the controls. Each value is the mean ± s.d. of three independent samples, carried out in duplicate. c: β-gal(RNAi); mt-mmpA(RNAi): Dj-mt-mmpA(RNAi). (TIF) [file pone.0055649.s010.tif]

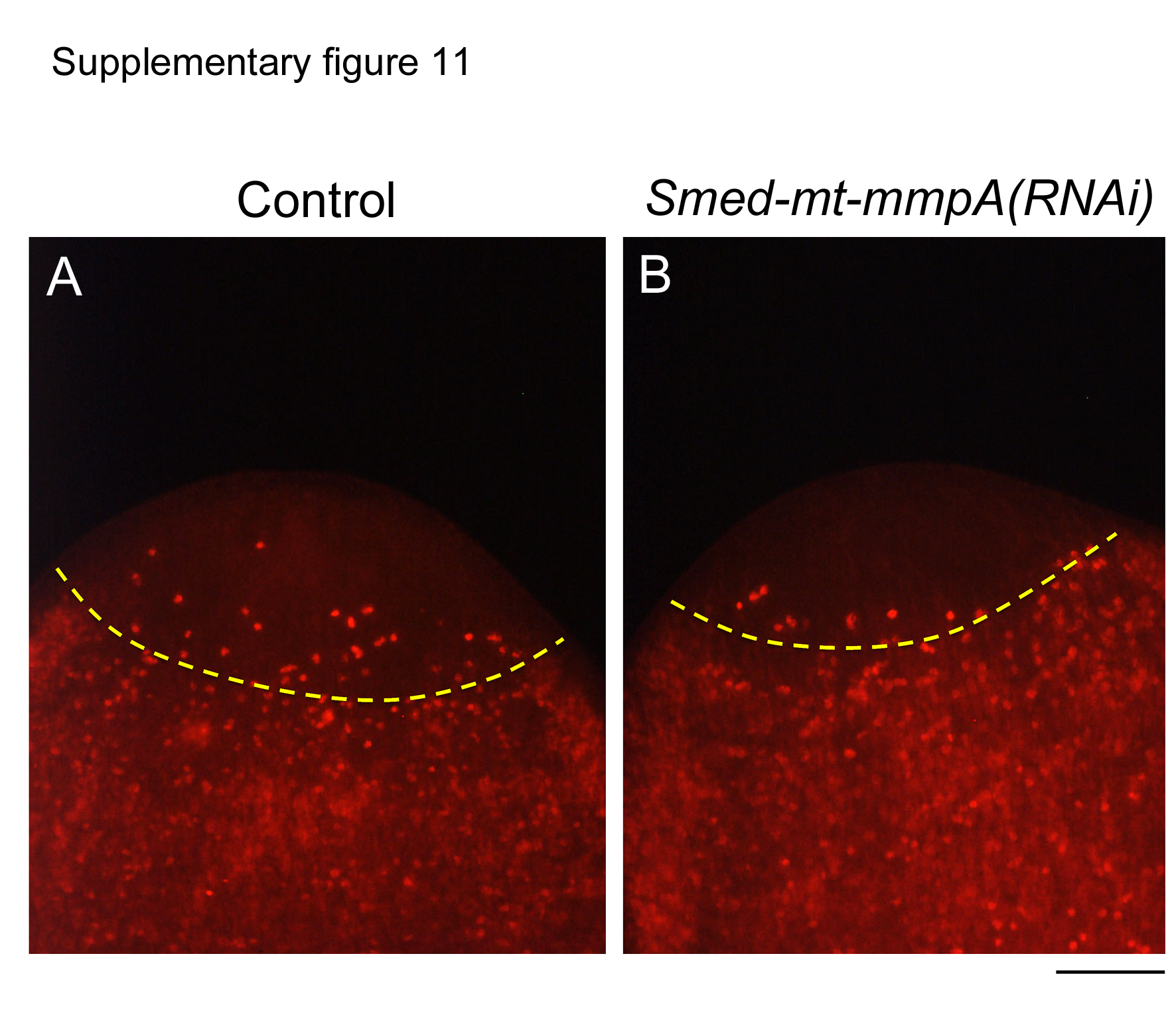

Supplement: Figure S11 — Immunostaining with anti-H3P antibody in Smed-mt-mmpA(RNAi) fragments, at 6 days of head regeneration. (A) β-gal(RNAi) control. (B) Smed-mt-mmpA(RNAi). After Smed-mt-mmpA(RNAi) silencing the regenerative blastema results reduced in size and a smaller number of anti-H3P-positive cells migrated into the blastema with respect to the control. Scale bar: 200 µm. (TIF) [file pone.0055649.s011.tif]
